# Ammonia Gas Sensor Fabricated by Multifunctional ZnO/GO Nanocomposites for Long‐Term, Self‐Powered Monitoring

**DOI:** 10.1002/advs.202516833

**Published:** 2025-12-05

**Authors:** Xingwei Wang, Likun Gong, Xiaohong Zhou

**Affiliations:** ^1^ State Key Laboratory of Regional Environment and Sustainability School of Environment School of Environment Tsinghua University Beijing 100084 China; ^2^ College of Science China University of Petroleum (East China) Qingdao Shandong 266580 China

**Keywords:** ammonia gas sensor, long‐term stability, supercapacitor, wearable TENG, ZnO/GO nanocomposites

## Abstract

As a promising hydrogen carrier for low‐carbon energy cycling, ammonia also represents the most abundant alkaline gas in the atmosphere, impacting environmental quality through diverse geophysical and chemical processes. Hence, developing NH_3_ sensing materials with high sensitivity and stability under self‐powered operation is essential. A one‐step in situ polymerization method is demonstrated to synthesize zinc oxide/graphene oxide (ZnO/GO) nanocomposites, serving as a gas‐sensitive film for ammonia sensing and as high‐performance electrode materials in supercapacitors, simultaneously. For the supercapacitor, the specific capacitance of 131 F g^−1^ at 1 A g^−1^ is achieved. The ammonia sensor featured a low detection limit (0.1 ppm) and fast response/recovery time (17 s/26 s @ 10 ppm NH_3_), surpassing standards set by the US Occupational Safety and Health Administration (50 ppm), while outperforming commercial NH_3_ gas sensors. By integrating the sensor into a detection instrument for fixed‐point monitoring, the response relative standard deviation of below 1% over 210 days of continuous testing is achieved. In addition, a wearable contact‐separated TENG is developed to harvest mechanical energy from a contact‐separation setup that mimicked human footsteps, achieving a maximum output power of 4.1 mW to directly drive the ammonia gas sensor. The multi‐scenario applications enhanced the spatial coverage and operational flexibility of NH_3_ concentration monitoring.

## Graphical abstract

Zinc oxide/graphene oxide (ZnO/GO) nanocomposites serve as a gas‐sensitive film for ammonia sensing and as high‐performance electrode materials in supercapacitors, simultaneously. A wearable contact‐separated triboelectric nanogenerator is designed to directly drive the ZnO/GO nanocomposites‐based ammonia sensor. Hence, different application scenarios are designed for a long‐term fixed‐type and self‐powered ammonia monitoring system, enhancing the spatial coverage and operational flexibility.

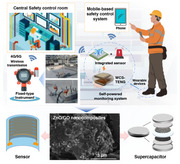

## 1 Introduction

Using ammonia as a hydrogen carrier presents a promising avenue for low‐carbon cycling to tackle the energy crisis.^[^ ^1^ ^]^ This development underscores the pressing need for effective monitoring and early warning systems to detect potential ammonia leaks in zero‐carbon vessels.^[^ ^2^ ^]^ The Occupational Safety and Health Administration sets the occupational exposure limit for ammonia at 50 ppm, highlighting the importance of maintaining safe concentrations in operational environments. Furthermore, ammonia, as the predominant alkaline gas in the atmosphere, exerts detrimental impacts on environmental quality.^[^ ^3^, ^4^ ^]^ Its presence facilitates the formation of secondary particles in the atmosphere, impairing air visibility, posing a potential threat to human health. The escalation of air pollution, accelerated soil acidification, and disruption of sensitive ecosystems due to ammonia emissions^[^ ^5^, ^6^, ^7^ ^]^ necessitate the development of sensitive, low‐power consumption, and environmentally friendly monitoring systems for tracking ammonia gas concentrations. However, the deployment of numerous ammonia gas sensing nodes presents challenges, including the need for highly sensitive, low‐power, and environmentally friendly sensing materials, as well as concerns regarding battery pollution and maintenance.^[^ ^2^ ^]^ Leveraging renewable energies harvested from the natural environment to facilitate self‐powered operation of monitoring networks is regarded as a sustainable alternative in contrast to the conventional grid‐based and battery‐powered approaches.^[^ ^8^, ^9^, ^10^ ^]^ In this context, developing NH_3_ sensing materials with excellent sensitivity, selectivity, and stability under self‐powered conditions is crucial for ensuring reliable and continuous monitoring.

The zinc oxide (ZnO) materials among transition metal oxides have received significant attention in energy storage and sensor devices due to their excellent electrochemical stability, high electrical conductivity, and favorable optical, electrical, and catalytic properties. However, in certain applications, pristine ZnO materials may face challenges such as low surface area and poor structural stability, which limit their performance.^[^ ^11^, ^12^ ^]^ To overcome these challenges, researchers have explored the combination of ZnO with other materials. 2D nanomaterials, with their high surface‐to‐volume ratio and increased carrier density, hold promise as foundational materials for innovative energy storage devices and sensing devices.^[^ ^13^, ^14^ ^]^ Graphene oxide (GO), as one of the earliest reported star 2D nanomaterials, has been used as an efficient, high‐performance electrode material for supercapacitors.^[^ ^15^, ^16^ ^]^ The combination of ZnO with GO can create a synergistic effect, enhancing electron transport, increasing the specific capacitance of the electrode material, and optimizing the sensitivity and selectivity of sensors.^[^ ^17^ ^]^ Moreover, the zeolitic imidazolate framework (ZIF)‐based structure of ZnO brings additional advantages, such as a high surface area and good chemical stability, as well as the tuning of ZnO's morphology and particle size, hence further improving its performance in supercapacitors and sensors. Therefore, ZIF‐derived ZnO/GO nanocomposites, owing to the porosity of the ZIF structure, the electrochemical activity of ZnO, and the high conductivity of GO, hold great potential for applications in supercapacitors.^[^ ^18^, ^19^, ^20^ ^]^ Simultaneously, the high surface‐to‐volume ratio and abundant active reaction sites of ZnO/GO nanocomposites with advantages in gas molecule adsorption‐desorption also make it a unique sensing material for the preparation of gas‐sensitive electrodes.^[^ ^21^, ^22^ ^]^ Despite the above extensive efforts, research on concise and low‐cost synthesis processes of ZnO/GO nanocomposites and further exploring them as dual‐functional materials for sensing and energy storage is still largely unexplored. In addition, Triboelectric nanogenerators (TENGs), driven by friction‐induced charging and electrostatic induction, have received considerable attention owing to their versatility in deployment and capacity for low‐frequency operation in self‐powered monitoring applications.^[^ ^23^, ^24^ ^]^ The advancement of wearable TENG technologies has become a prominent direction in energy harvesting research, as their inherent wearability provides a strategic advantage for harnessing the ubiquitous mechanical energy arising from human motion.^[^ ^25^, ^26^ ^]^

Inspired by the above research and to address these bottlenecks, we proposed a one‐step in situ polymerization method to synthesize low‐cost zinc‐doped carbon nanocomposites, i.e., ZnO/GO nanocomposites, which then served as a gas‐sensitive film for ammonia sensing and as high‐performance electrode materials in supercapacitors, simultaneously. The working principle and performance of the ZnO/GO nanocomposite‐based ammonia sensor and supercapacitor were systematically investigated. Two application scenarios were further explored, including an ammonia gas detector designed for fixed‐point monitoring and a portable system integrated with a footwear‐incorporated wearable TENG, in which the biomechanical energy generated during walking was harnessed for self‐powered ammonia sensing. Finally, the long‐term stability of the developed ammonia sensor was experimentally validated. The superior reliability and application potential of the developed system provide new opportunities for advancing sustainable energy and environmental technologies.

## 2 Results

### 2.1 Characterization and Simulation Calculation of ZnO/GO Nanocomposites

**Figure 1:**
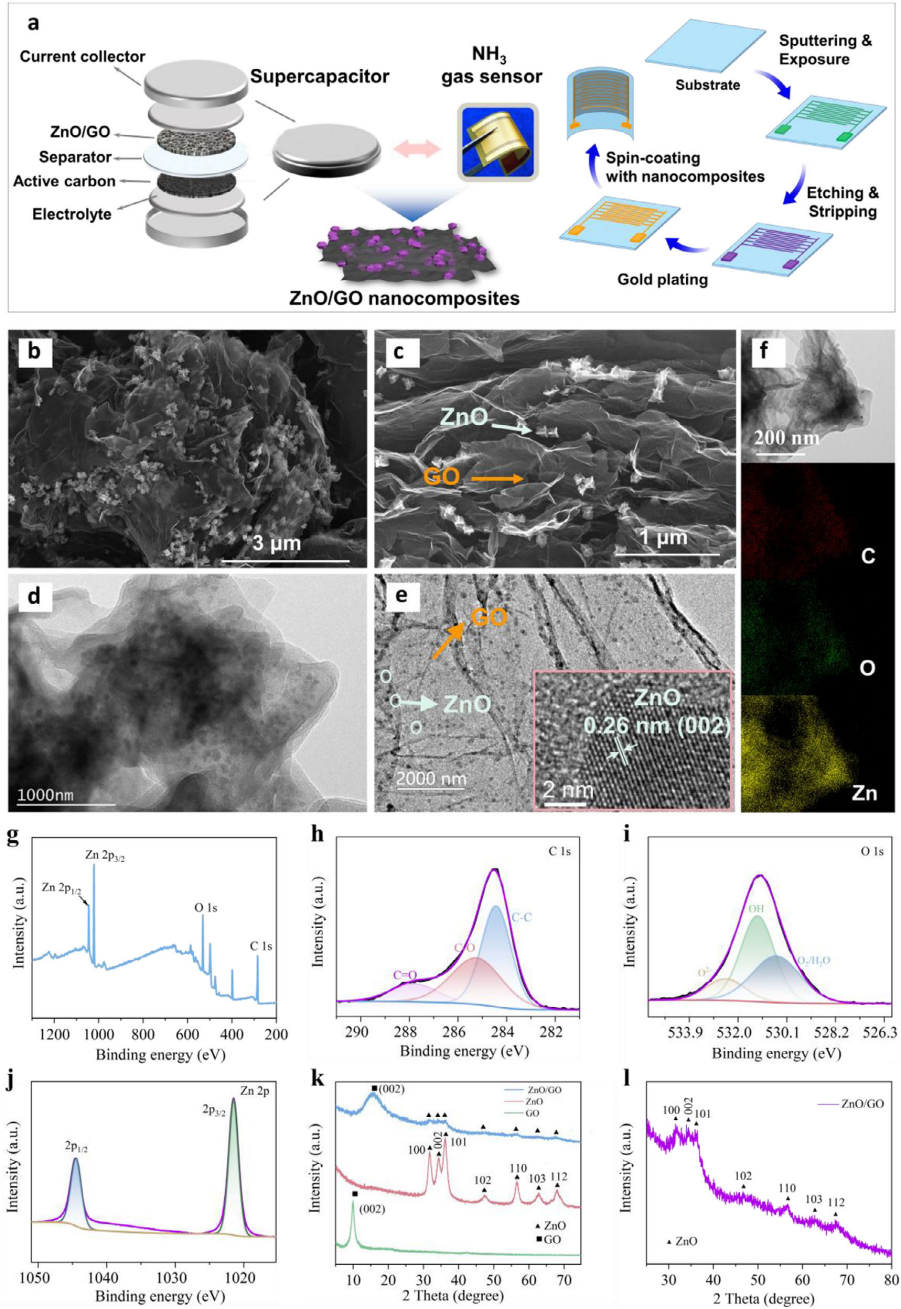
Characterization of ZnO/GO nanocomposites. a) Schematic diagram of the preparation of ZnO/GO nanocomposites simultaneously used for the fabrication of supercapacitors and gas sensors. b, c) SEM and d, e) TEM image of the synthesized ZnO/GO nanocomposite. f) EDX elemental mapping of ZnO/GO nanocomposites. XPS spectra of ZnO/GO nanocomposites: g) Survey scan, h) C 1s, i) O 1s, j) Zn 2p, k) XRD patterns of ZnO, GO, and ZnO/GO nanocomposites. l) Amplified XRD patterns of ZnO/GO nanocomposites.

We synthesized the ZnO/GO nanocomposites using a one‐step in situ polymerization method (Figure S1, Supporting Information), which then served as a gas‐sensitive film for ammonia sensing and as high‐performance electrode materials in supercapacitors, simultaneously (**Figure** **1**a). The detailed information of the synthesized materials via the in situ polymerization method can be found in the Experiment section. The unique spherical nanostructure of the ZnO nanomaterials was evident in the Scanning Electron Microscopy (SEM) image, as shown in Figure S2a (Supporting Information), with a size of ≈100 nm. The irregular sheet structure of GO nanomaterials endowed them with distinctive electrochemical properties (Figure S2b, Supporting Information). The 2D structure of GO enhanced the dispersity of ZnO nanoparticles and the interface with gas molecules concurrently. Hence, it reduced the charge transfer distance, amplifying the response to gas molecules.^[^ ^27^ ^]^ The elemental mapping images of the ZnO/GO nanocomposites obtained by Energy‐Dispersive X‐ray Spectroscopy (EDS) demonstrated a uniform distribution of C, O, and Zn elements, confirming the homogeneous composition of ZnO and GO (Figure S3, Supporting Information). In addition, a small amount of N element was detected in the elemental mapping, and the corresponding N peak was also observed in the EDS spectrum (Figure S4, Supporting Information). Quantitative analysis showed that the mass fraction of N in the ZnO/GO nanocomposites was ≈9.118%, which was much lower than that of Zn (44.657%) (Table S1, Supporting Information). The presence of N might be attributed to the incomplete decomposition of the precursor C_8_H_12_N_4_Zn (ZIF‐8) during the calcination process, resulting in a small amount of N‐doped carbon residue or N‐doping defects within the ZnO material. Figure 1b,c demonstrated the successful agglomeration of ZnO nanoparticles and GO sheets, forming the ZnO/GO nanocomposite material. The internal structures of ZnO (Figure S5a, Supporting Information) and GO (Figure S5b, Supporting Information) were examined using Transmission Electron Microscopy (TEM). Ultra‐thin, transparent GO nanosheets demonstrated a wrinkled structure. High‐Resolution TEM (HRTEM) images (Figure 1d,e) showed numerous ZnO clusters evenly distributed on the surface of GO, consistent with the SEM findings. These Zn nanospheres increased the number of active sites on the surface of GO, thereby enhancing the adsorption potential of gas molecules and the electrochemical reaction activity. The lattice distance of 0.26 nm depicted in HRTEM images was attributed to the (002) crystal plane of ZnO.^[^ ^28^ ^]^ Figure 1f featured an elemental mapping image of C, O, and Zn, showing the uniform distribution of varied elements on the ZnO/GO nanocomposites.

The chemical bonds in the ZnO/GO nanocomposites were analyzed through X‐ray Photoelectron Spectroscopy (XPS). The full XPS spectrum of the ZnO/GO nanocomposites indicated the presence of C, O, and Zn elements (Figure 1g). As similar as revealed by EDS, the N 1s peak (400 eV) was also present might be due to the incomplete decomposition of ZIF‐8 during calcination, resulting in a small amount of N‐doped carbon residues or nitrogen‐doped defect sites. The C1s XPS spectrum of the ZnO/GO nanocomposites was fitted into three peaks (Figure 1h).^[^ ^29^ ^]^ The first peak was the C═O bond of C Sp^2^. The second peak was the C─O bond formed by carbonyl carbon with epoxide/alkoxy and the third peak was the C─C bond of C Sp^2^.^[^ ^30^ ^]^ Figure 1i depicted the high‐resolution XPS spectrum of O1s, which was fitted into three peaks with binding energies of 532.8, 529.8 and 531.2 eV, attributed to O^2−^ oxygen ions, OH hydroxyl, and O_2_/OH oxygen/hydroxyl, respectively.^[^ ^31^ ^]^ The XPS spectrum of Zn 2p (Figure 1j) was fitted into two peaks at binding energies of 1023.1 and 1046.9 eV, representing the Zn 2p_3/2_ and Zn 2p_1/2_ orbital states of Zn^2+^ ions, respectively.^[^ ^32^ ^]^ Such findings signal the successful synthesis of the ZnO/GO nanocomposites. The formation, phase purity, and crystal structure of the samples were examined using XRD, as shown in Figure 1k. The diffraction graph of ZnO comprised intensely sharp peaks, attributed to the crystalline nature of nanostructures. The broad diffraction peak at 27° was ascribed to the (002) crystal facet of GO.^[^ ^33^ ^]^ Peaks appearing at 31.7°, 34.5°, 36.25°, 47.6°, 56.6°, 62.9°, and 67.9° were attributed to the (100), (002), (101), (102), (110), (103) and (112) crystal faces of hexagonal crystal‐structured ZnO, as shown in Figure 1l. The location and intensity of diffraction peaks were consistent with those in the JCPDS card (number 36–1451).^[^ ^34^ ^]^ Notably, in the ZnO/GO nanocomposites, the diffraction peaks of ZnO and GO were distinctly evident, indicating successful integration of ZnO into GO. As further evidenced by the Raman spectra of ZIF‐8, ZnO, GO, and the ZnO/GO nanocomposites (Figure S6, Supporting Information), the ZnO sample exhibited a characteristic A_1_ (LO) mode at 565 cm^−1^, with a slight red shift attributed to N‐doping–induced lattice distortion.^[^ ^35^ ^]^ In contrast, the Raman features of the ZIF‐8 precursor differed from those of ZnO, confirming its successful conversion into ZnO during calcination. GO showed the typical D, G, and 2D bands at 1342, 1573, and 2700 cm^−1^, respectively.^[^ ^36^ ^]^ The ZnO/GO nanocomposites displayed characteristic peaks of both ZnO and GO, further verifying the successful formation of the hybrid nanostructure. Figure S7 (Supporting Information) showed the N_2_ adsorption of ZnO, GO, and ZnO/GO. The adsorption capacity of N_2_ in ZnO/GO was higher than that of pure ZnO and GO samples, demonstrating the enhanced solubility of N_2_ in the nanocomposites.

**Figure 2:**
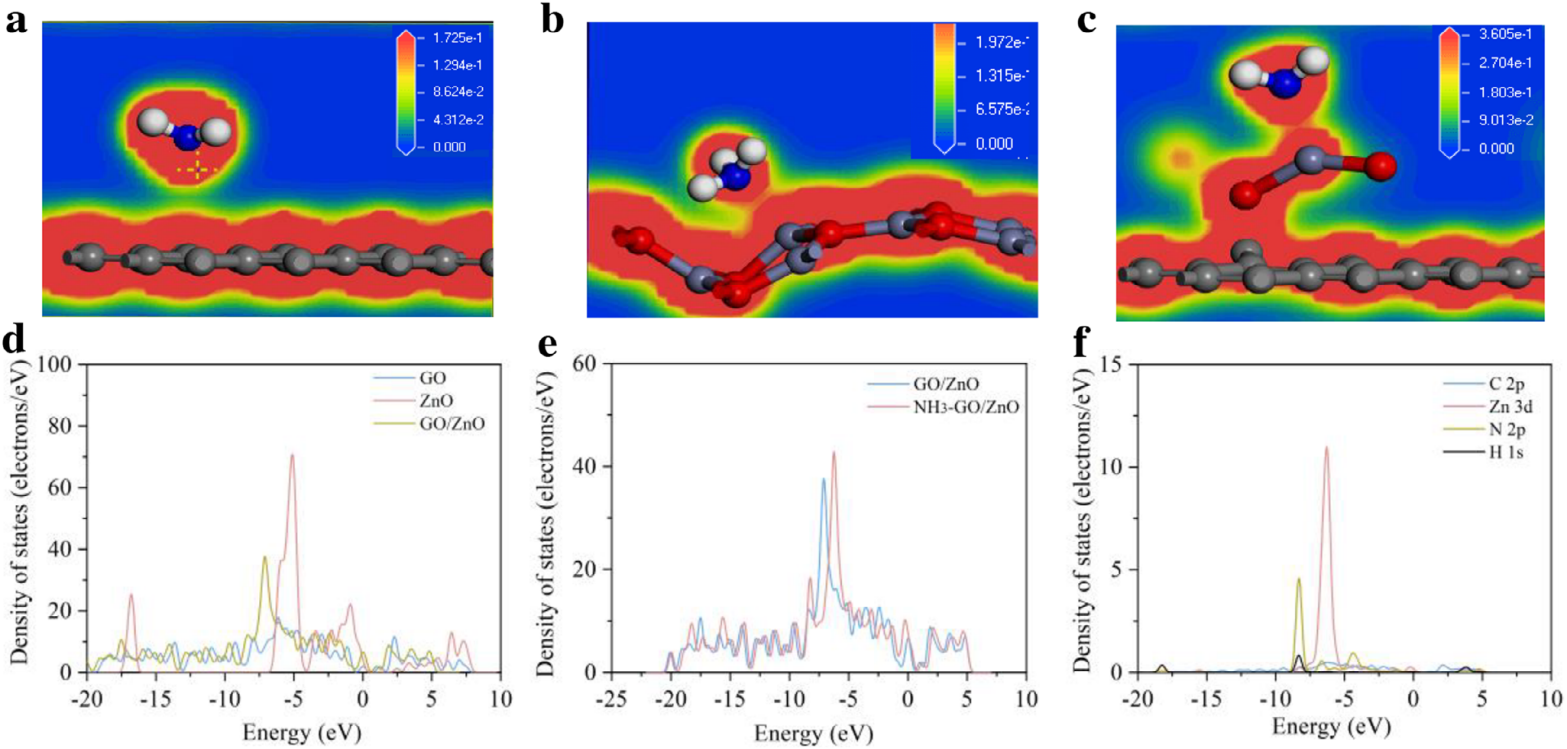
Density functional theory (DFT) simulation of ZnO/GO nanocomposites. Charge density of a) GO, b) ZnO, and c) ZnO/GO after NH_3_ molecule adsorption (black: C atoms, blue: N, red: O, grey: Zn, white: H). d, e) TDOS diagrams of the ZnO/GO system before and after adsorption of NH_3_ molecules. f) PDOS diagrams of ZnO/GO system adsorption of NH_3_ molecules.

To further validate the feasibility of the Zn‐C composite material as a gas‐sensitive film, we employed Density Functional Theory (DFT) for geometric optimization calculations. The comparative absorption of ZnO, GO, and ZnO/GO on NH_3_ was analyzed utilizing geometric optimization computations. These computations were executed via the DMol3 code incorporated within the Materials Studio 8.0 software package. Figure S8 (Supporting Information) exhibited the molecular model diagrams for the adsorption of NH_3_ on the surfaces of GO, ZnO, and ZnO/GO. The simplified structure of ZnO/GO was obtained through a planar cutting operation. Charge density maps of GO, ZnO, and ZnO/GO with adsorbed NH_3_ were displayed in **Figure** **2**a–c. The GO material exhibited a low affinity for NH_3_. In contrast, ZnO and ZnO/GO exhibited a stronger chemical bond with NH_3_. The charge transfer in the ZnO/GO system demonstrated enhanced strength compared to that in the ZnO system.^[^ ^37^ ^]^ Optimized geometric parameters, such as bond angles and lengths of NH_3_, were presented in **Table** **1**. Additionally, the shortest distance between NH_3_ and the material surface was provided. The adsorption energy of materials was calculated using the following equation:

(1) $$E_{\mathrm{ad}}=E_{\mathrm{gas}/\mathrm{sub}}-E_{\mathrm{sub}}-E_{\mathrm{gas}}$$

Where E_gas/sub_ is the energy of the entire system; E_sub_ is the energy of the GO, ZnO, or ZnO/GO; and E_gas_ is the energy of an NH_3_ molecule.

**Table 1:** The parameters of pristine GO, ZnO, and GO/ZnO adsorption systems.

Systems	Bond length of N─H [Å]	Bond angle of H─N─H [^o^]	Distance [Å]	E_ad_ [eV]
GO	1.020	107.499	2.231	−1.388
ZnO	1.023	105.478	3.237	−0.218
ZnO/GO	1.026	105.865	2.155	−1.878

As shown in Table 1, the adsorption energy (E_ad_) of ZnO/GO was 1.878 eV, which surpassed that of GO and ZnO. Consequently, ZnO/GO nanocomposites exhibited superior NH_3_ adsorption capability compared to GO and ZnO.

Total Density States (TDOS) and Projected Density States (PDOS) of GO, ZnO, and ZnO/GO were analyzed to expose the electronic structures. As shown in Figure 2d, electronic density curves of ZnO/GO in the TDOS graph shifted to the left as compared to that of GO, and a new peak emerged at −5.2 eV due to the influence of the Zn 3d orbital. After NH_3_ adsorption, as shown in Figure 2e, peaks in the TDOS graph were enhanced near −5.7, −8.1, and −15.5 eV. The changes in the TDOS graph, as shown in Figure S9 (Supporting Information), were primarily attributed to the interaction among Zn 3d orbitals, C 2p orbitals, and O 2p orbitals. It was observed in Figure 2f that these orbitals had obvious overlapping peaks at −8.2 and −6.6 eV. This indicates strong hybridization between the NH_3_ molecule and ZnO/GO nanocomposites.^[^ ^38^ ^]^

### 2.2 Performance Evaluation of Supercapacitors and Sensors Based on ZnO/GO Nanocomposites

**Figure 3:**
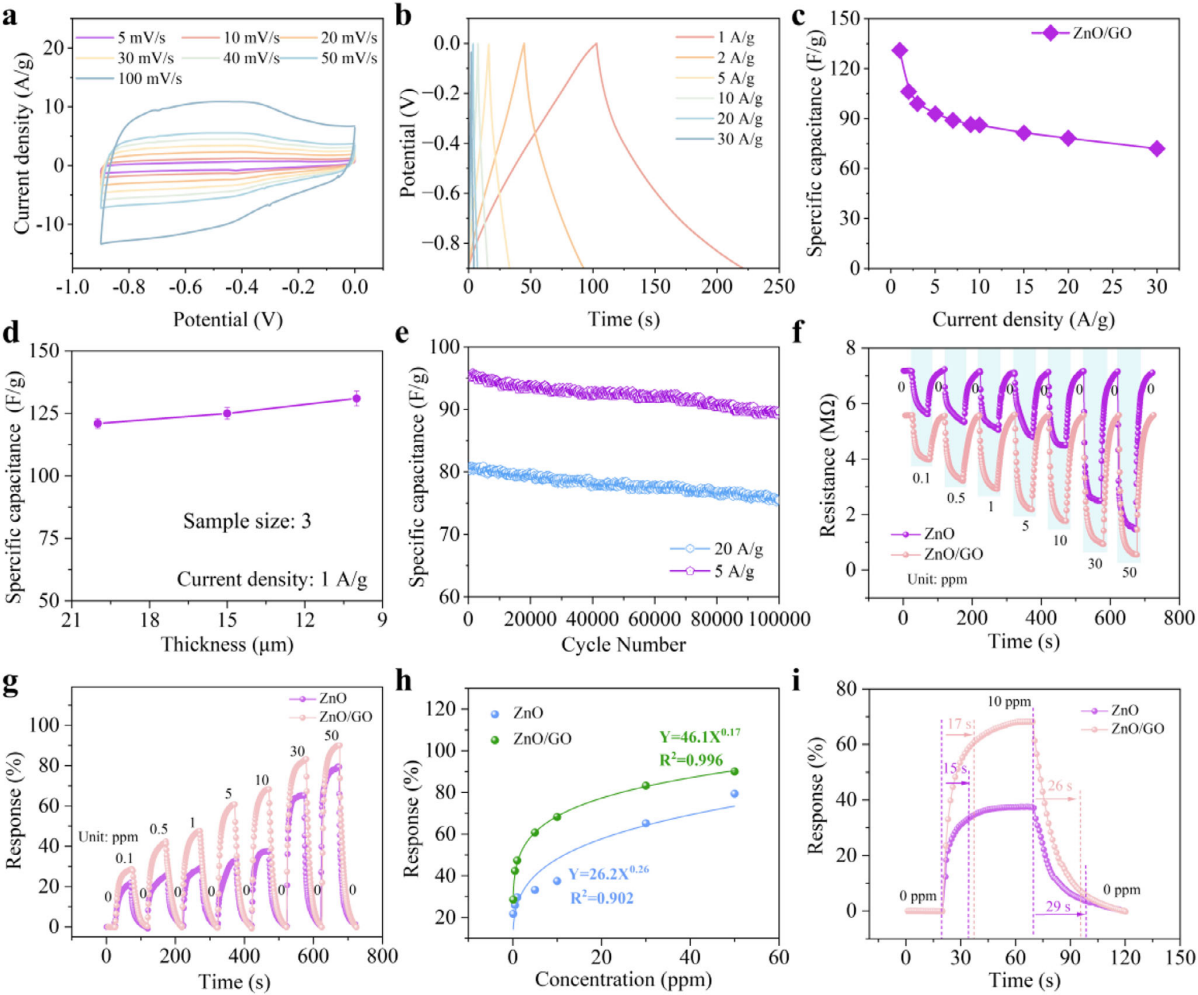
Evaluation of supercapacitor and gas‐sensing performance. a, b) CV and GCD curves of the ZnO/GO electrode. c) Specific capacitance of ZnO/GO electrode at different current densities. d) Specific capacitance of ZnO/GO‐based supercapacitors fabricated with different thicknesses of electrode materials at a current density of 1 A g^−1^. e) Evaluation of the durability of the specific capacitance of the supercapacitor. f) The resistance changes of NH_3_ gas sensors based on pure ZnO and ZnO/GO composite under different NH_3_ concentrations at 20 °C. g) The response of NH_3_ gas sensors based on pure ZnO and ZnO/GO composite under different NH_3_ concentrations at 20 °C and h) Response fitting curves. i) The response/recovery characteristic curves at NH_3_ concentration of 10 ppm at 20 °C.

In a three‐electrode system with 6 m KOH as the electrolyte, the electrochemical behavior of ZnO/GO was characterized using techniques such as Cyclic Voltammetry (CV), Galvanic Charge/Discharge (GCD), and Electrochemical Impedance Spectroscopy (EIS). The CV curves of ZnO/GO at varying scan rates from 5 to 100 mV s^−1^ were depicted in **Figure** **3**a, which displayed the bidirectional capacitive behavior of carbon materials. As shown in Figure 3b, the specific capacitance of the ZnO/GO electrode was calculated using GCD, revealing that the electrode had a specific capacitance of 131 F g^−1^ at a current density of 1 A g^−1^. Even when the current density increased to 50 A g^−1^, the ZnO/GO electrode maintained a capacitance of 62.2 F g^−1^, achieving a capacitance retention efficiency of 47.5% (Figure 3c). From the Ragone plot of the ZnO/GO‐based supercapacitor under different testing conditions (Figure S10, Supporting Information), it was observed that the energy density gradually decreased with increasing power density, reflecting the trade‐off between energy storage capability and power delivery. The device exhibited a maximum energy density of 14.7 Wh·kg^−1^ at a power density of 450 W·kg^−1^, whereas a high power density of 13.5 kW·kg^−1^ was achieved at an energy density of 8.1 Wh·kg^−1^. The smaller the areal density of the electrode, the larger the area in contact with the electrolyte. Thinner electrodes not only increased the specific surface area but also shortened the longitudinal transport path of electrolyte ions, thereby improving the charging and discharging rates of the capacitor. At a current density of 1 A g^−1^, we tested the rate performance of electrodes with different thicknesses of 20, 15, and 10 µm. The thinner the electrode, the better the rate performance. As shown in Figure 3d, the rate performance of the capacitor improved as the thickness decreased. However, considering material density and cost‐effectiveness, we ultimately selected a thickness of 10 µm. Video S1 (Supporting Information) provided further validation of its outstanding performance by demonstrating the operational efficacy of four supercapacitors in series (0.9×4 = 3.6 V) in charging mobile phones. Impressively, after 100 000 charge/discharge cycles, the specific capacitance of the supercapacitor retained ≈94% of its initial value at both 5 and 20 A g^−1^ (Figure 3e), demonstrating the durability/stability of the supercapacitor system.

**Figure 4:**
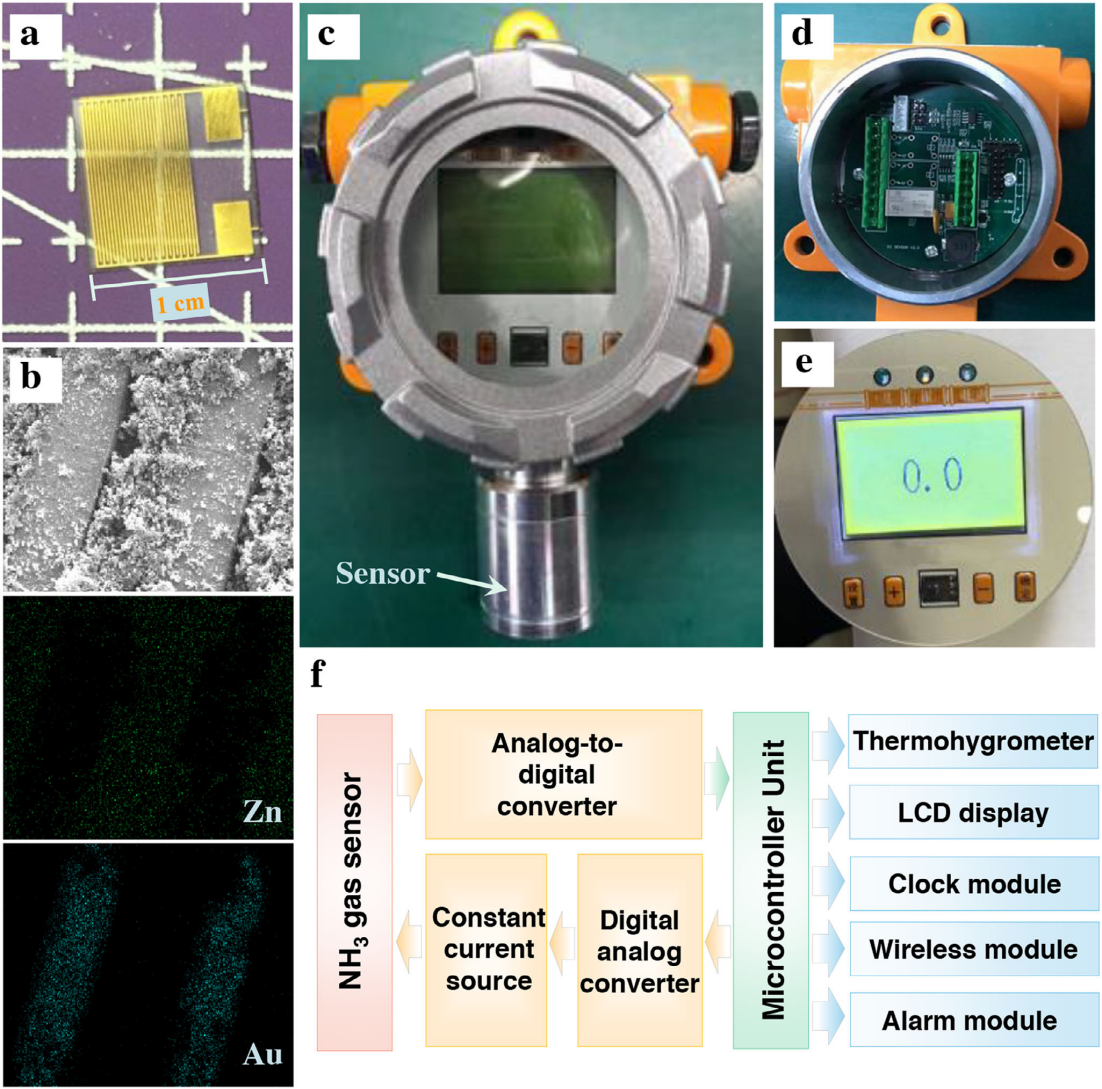
Design and implementation of a ZnO/GO‐based ammonia detection instrument. a) Photograph of the interdigital electrode for deposition of ZnO/GO nanocomposites. b) SEM images and elemental mapping of the ZnO/GO nanocomposites‐deposited sensor prepared with the optimized spin‐coating time of 60 s. Photographs of the integrated ammonia gas detector and its functional modules: c) external view, d) internal PCB module, and e) display screen. f) Schematic illustration of the system architecture of the ammonia gas detector.

Gas sensors were sequentially placed within dry NH_3_ of various concentrations. When a certain concentration of NH_3_ resulted in steady sensor resistance, the sensor was transitioned back into the air. This reset the resistance of the gas sensor to its initial state before being placed into the next concentration of NH_3_. Figure 3f illustrates the resistance changes of ZnO and ZnO/GO gas sensors at NH_3_ concentrations of 0.1, 0.5, 1, 5, 10, 30, and 50 ppm. We tested ammonia gas sensors with different film thicknesses formed under spin‐coating times of 30, 60, and 120 s, in response to a 5 ppm NH_3_ concentration (Figure S11, Supporting Information). Results showed the sensor with a 60 s spin‐coating time had the best response, so this duration was used for further experiments. SEM and elemental mapping images of sensors with the three spin‐coating times are shown in Figures 4b and S12 (Supporting Information), revealing a more uniform film distribution on the sensor surface at a 60 s spin‐coating time. As NH_3_ concentration increased, the resistances of both ZnO and ZnO/GO gas sensors dropped. However, with the increase of NH_3_ concentration, the resistance of the GO gas sensor resistances escalated (Figure S13, Supporting Information). The doping of GO reduced the resistance of sensors, thus benefiting resistance signal detection and subsequent hardware circuit design.

The response (S) of sensors was defined as:

(2) $$S=|R_{a}-R_{g}|/R_{a}\;\times \;100\%$$

Where R_a_ is the initial resistance value of the sensors in the air, R_g_ is the resistance value of the sensors in the presence of NH_3_ gas.

The responses of ZnO and ZnO/GO‐based sensors were presented in Figure 3g. The integration of GO escalated the response to NH_3_ relative to ZnO alone. We prepared ZnO/GO composites with different molar ratios and tested the sensor response at an NH_3_ concentration of 5 ppm (Figure S14, Supporting Information). As the molar ratio of ZnO increased, the sensor response first increased and then decreased. The highest response was obtained at a ZnO molar ratio of 66.7%, which was attributed to the optimal synergistic effect between ZnO and GO materials. The fitting functions for the ZnO and ZnO/GO‐based sensors were denoted as Y = 46.1X^0.17^ and Y = 26.2X^0.26^, respectively, displaying fitting coefficients of 0.99 and 0.90. A saturation tendency was observed as the NH_3_ concentration gradually increased to 30 ppm (Figure 3h). The response time was defined as the duration required for the sensor signal to reach 90% of its total change upon exposure to the target, whereas the recovery time was defined as the period needed for the signal to decrease from its peak value to 10% of that peak. Figure 3i exhibited the response/recovery curves at an NH_3_ concentration of 10 ppm. The recovery time for the ZnO/GO‐based sensor totaled 26 s, demonstrating a shorter duration than that of the ZnO‐based sensor. We further tested seven concentration levels of ammonia gas (0.1, 0.5, 1, 5, 10, 30, and 50 ppm) using both gas chromatography and our sensor (Figure S15, Supporting Information). The experimental results demonstrated a high level of consistency between the two methods across all concentrations, with a relative deviation of less than 2%. This indicated that our device showed excellent accuracy at different ammonia concentrations.

### 2.3 Integration of the Ammonia Gas Sensor into a Detection Instrument

As detailed in Experimental Section, the ammonia gas sensor had dimensions of ≈1 cm × 1 cm, with both the line spacing and the width of the interdigital electrodes being 75 µm (**Figure** **4**a). The ZnO/GO nanocomposite was deposited onto the interdigital electrode via the spin‐coating method with the optimized spin‐coating time of 60 s, which was uniformly filled the gap between interdigital electrodes revealed with the SEM image and elemental mapping (Figures 4b; S12, Supporting Information).

The sensor was further integrated into an ammonia gas detector equipped with a display screen and control module. To meet practical requirements such as explosion protection, the detector was fabricated with the sensor encapsulated in an explosion‐proof housing (Figure 4c). The signal acquisition, communication, and display circuits were developed using printed circuit board (PCB) technology (Figure 4d). When activated, the display presented the real‐time ammonia concentration (Figure 4e). As illustrated in Figure 4f, the resistance signal from the sensor was transmitted to the microcontroller unit (MCU) via a digital–analog converter, while the MCU provided a constant current source to the sensor. A thermometer–hygrometer connected to the MCU enabled automatic calibration based on ambient temperature and humidity. When the detected ammonia concentration exceeded the preset threshold, the MCU triggered audible and visual alarms for early warning. Photographs of the circuit modules before and after assembly are shown in Figure S16 (Supporting Information).

### 2.4 Stability and Environmental Endurance Testing of the Ammonia Gas Detector

**Figure 5:**
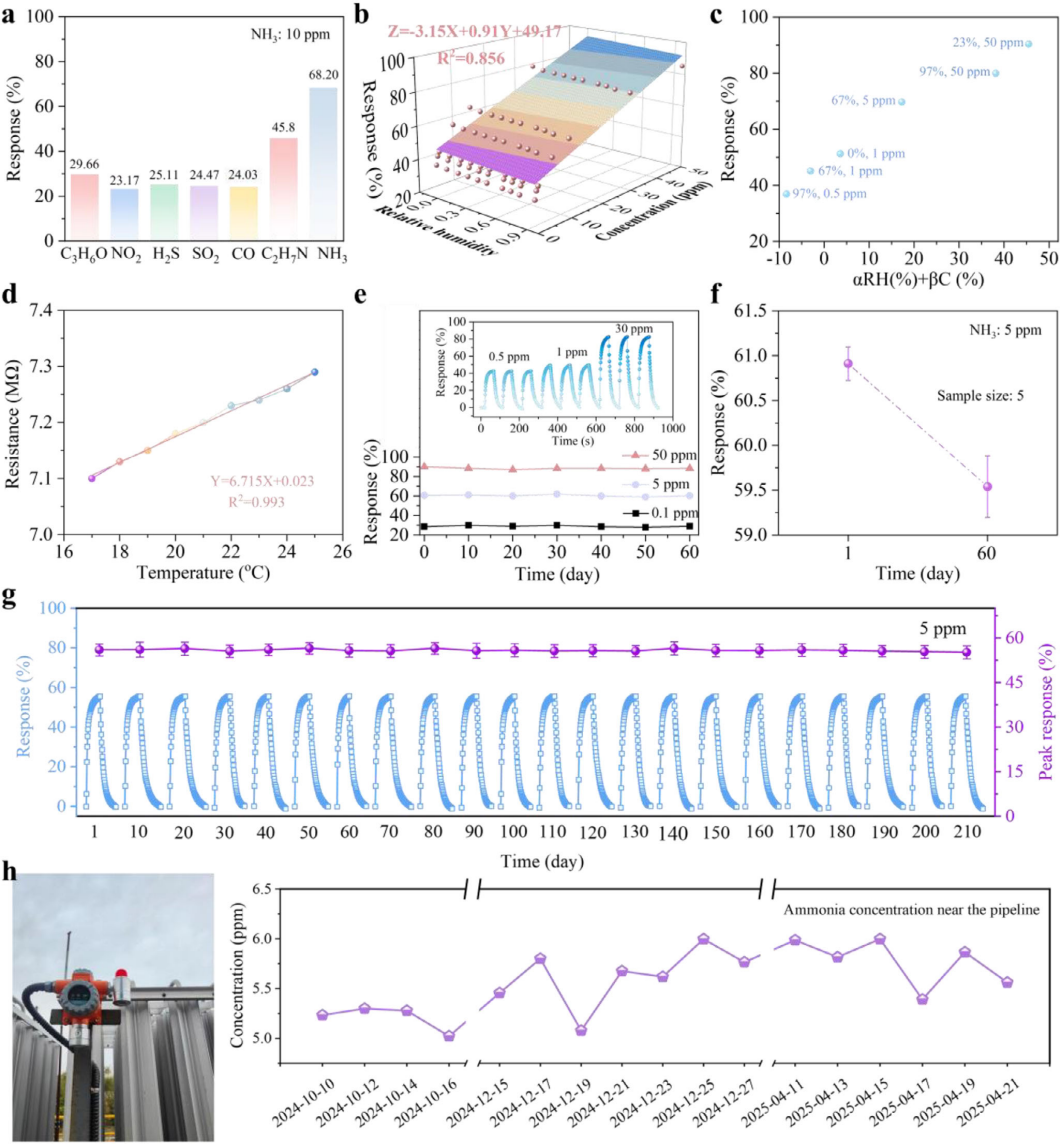
Environmental endurance and stability tests of the ammonia gas detector. a) Selectivity of the ZnO/GO composite‐based sensor. b) 3D scatter of the response vs both humidity and NH_3_ concentration for the ZnO/GO‐based sensor. c) Linear behavior of the response vs both humidity and NH_3_ concentration for the ZnO/GO‐based sensor. d) The temperature effect on the ZnO/GO‐based sensor. e) Comparison of response/recovery time between this work and other types of NH_3_ gas sensors. f) Repeatability and stability measurement results of the ZnO/GO‐based sensor under different NH_3_ concentrations. g) Long‐term stability of ZnO/GO‐based sensors over 210 days under exposure to 5 ppm NH_3_. h) Photograph of the installed ammonia detector and six months of NH_3_ concentration monitoring from October 10, 2024, to April 21, 2025.

**Figure** **5**a illustrates the selectivity of the ZnO/GO‐based gas detector toward various gases at 10 ppm, including acetone, nitrogen dioxide, hydrogen sulfide, sulfur dioxide, carbon monoxide, ethylamine, and ammonia. The detector exhibited a significantly higher response to NH_3_ than to the other interfering gases, demonstrating its excellent selectivity toward ammonia. To investigate the effects of relative humidity (RH) on the performance of the sensor, we prepared saturated salt solutions with various concentrations to emulate an RH range of 0–97%. As depicted in Figure S17 (Supporting Information), a decrease in the base resistance of sensors was observed as the RH increased, which might be attributed to the enhancement in ion conductivity resulting from the competitive adsorption of water molecules on the sensing surface. The relationship between the sensor response, RH (0%–97%), and the NH_3_ concentration (0–50 ppm) was illustrated in a 3D coordinate system in Figure 5b. We determined a regression equation, Z = −0.1X+3.34Y+49.17, exhibiting a regression coefficient R^2^ of 0.856. The association between sensor response (S), RH, and NH_3_ concentration (C) was expressed through the formula S = S_0_+αRH+βC. The estimated coefficients α, β, and S_0_ were −0.1, 3.34, and 49.17, respectively. We developed the fitted line in Figure 5c using αRH+βC as the independent variables and sensor response (S) as the dependent variable. The sensor response tended to decrease with the increase in RH. This trend was due to the occupation of active sites on the surface of sensing materials by water molecules under high humidity conditions. Figure 5d represented the influence of temperature on the base resistance of the ZnO/GO‐based sensors. The base resistance of the sensor showed minimal fluctuation within the temperature range of 17–25 °C, indicating that room temperature had little effect on the sensor. After measuring the response values of the NH_3_ sensor at different temperatures in the experiment, an additional temperature sensor can be installed on the integrated device during practical applications to calibrate the sensor's detection values.

Inset of Figure 5e showed the repeatability of the ZnO/GO‐based sensor at NH_3_ concentrations of 0.5, 1, and 30 ppm. After three cycles between air and NH_3_, the sensor's response reverted to its initial state, indicating its excellent repeatability. The sensor's response was also examined every 10 days for 60 days under NH_3_ concentrations of 0.1, 5, and 50 ppm, showing minimal variation. Moreover, Figure S18 (Supporting Information) illustrates the reproducibility of five ZnO/GO‐based ammonia sensors exposed to 5 ppm NH_3_ over a 60‐day period. The sensors exhibited consistent responses on both Day 1 and Day 60, with relative standard deviations (RSDs) of 0.34% and 0.65%, respectively, indicating good reproducibility and long‐term stability across different sensors (Figure 5f). In addition to stability, other performance, including the response value and response/recovery time of the five sensors exposed to 10 ppm NH_3_ was summarized in Table S2 (Supporting Information). The results indicated that five sensors exhibited good reproducibility across multiple comparison parameters. To further evaluate the long‐term stability of the ZnO/GO sensor, the response value of the sensor over the 210‐day period at the 5 ppm NH_3_ concentration was tested, and the RSD of the response signals measured every 10 days was 0.65%, indicating excellent reproducibility and long‐term stability of the sensor performance (Figure 5g). The integrated ammonia gas detector was installed next to the NH_3_ transmission pipeline to monitor the local NH_3_ concentration (Figure 5h). This fixed‐type instrument continuously measured the NH_3_ levels near the pipeline of an industrial park over a period of ≈6 months. During this time, the NH_3_ concentration remained around 5–6 ppm, in agreement with the actual conditions.

### 2.5 Self‐Powered Ammonia Gas Sensing by a Wearable TENG

**Figure 6:**
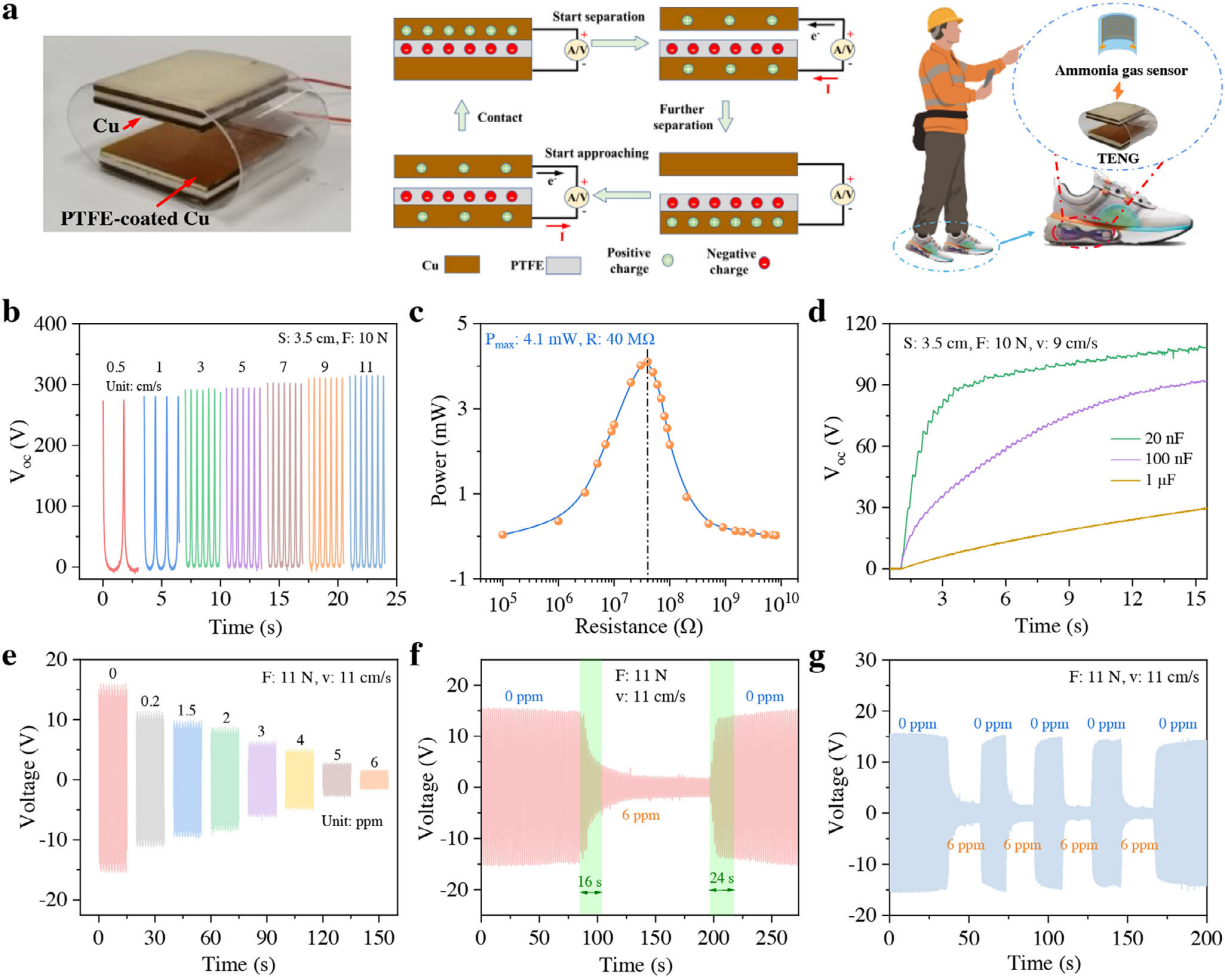
Self‐powered ammonia gas sensing by a wearable contact‐separated TENG (WCS‐TENG). a) The schematic diagram illustrating the structure, working principle, and application prospect of the WCS‐TENG. b) Effect of speed on open‐circuit voltage of WCS‐TENG. c) Variation curve of WCS‐TENG output power at different resistances. d) Capacitor charging curve of WCS‐TENG. e) The voltage response of the sensor at varying concentrations of NH_3_ gas. f) Voltage fluctuations of the sensor during NH_3_ concentration transitions between 0 and 50 ppm. g) Response and recovery time of the sensor under the WCS‐TENG‐driven operation.

The wearable contact‐separated triboelectric nanogenerator (WCS‐TENG), configured in a contact–separation mode, generated an alternating current voltage through the external circuit during operation (**Figure** **6**a). Copper (Cu) lost electrons and became positively charged, while polytetrafluoroethylene (PTFE) gained electrons and became negatively charged, and its mechanical motion emulated human stepping. During walking, the kinetic energy of the human body was converted into electrical power through the coupling effect of contact electrification and electrostatic induction, with PTFE and Cu serving as the triboelectric materials of the insole‐type device. The performance of the WCS‐TENG was evaluated using a standard contact–separation testing system capable of controlling the driving speed and frequency. As shown in Figure 6b, the open‐circuit voltage (V_oc_) of the WCS‐TENG was measured at different driving speeds. Under the fixed conditions with PTFE and Cu material spacing of 3.5 cm and an applied pressure of 10 N, the V_oc_ increased steadily from 0.5 to 11 cm s^−1^, demonstrating a clear dependence on driving speed. This enhancement was attributed to the higher frequency of contact‐separation cycles, which improved charge transfer, hence increasing the induced potential. Detailed performance parameters of the WCS‐TENG were shown in Note S1 (Supporting Information) (Figures S19–S28, Supporting Information). The maximum output power of 4.1 mW was achieved at an external resistance of 40 MΩ, calculated from the resulting peak‐to‐peak voltage and the short‐circuit current (Figure 6c), reflecting the load‐matching characteristics of the WCS‐TENG and indicating the presence of an optimal impedance for maximum power output. Further, to verify the energy storage capability of the WCS‐TENG, we used its output to charge capacitors at different capacitances (2077 nF, 100 nF, 1 µF) via rectification, voltage step‐down, and current step‐up modules. As shown in Figure 6d, the WCS‐TENG charged the 1 µF capacitor to 30 V in ≈15 s, demonstrating its potential in low‐frequency mechanical energy harvesting and micro‐energy storage systems.

Notably, since both the WCS‐TENG and the ZnO/GO‐based sensor have internal resistances in the MΩ range and the sensor features low power consumption, the WCS‐TENG harvesting energy from footsteps can directly power the sensor, allowing the NH_3_ concentration to be determined from the voltage across it. Therefore, we designed a self‐powered NH_3_ detection system, as illustrated in Figure S29 (Supporting Information), in which the sensor was coupled with a resistor (5 MΩ) and an LED indicator to enable threshold‐based alarm signaling. The drop in resistance caused by the increased NH_3_ gas concentration allowed a strong electrical current to flow through the circuit, powering the LED and causing it to illuminate brightly. This system thus provided a direct visual warning for NH_3_ concentrations exceeding the threshold. Using power from the WCS‐TENG, we tested the voltage of the ZnO/GO‐based sensor in the self‐powered detection system at NH_3_ concentrations ranging from 0 to 50 ppm. As the NH_3_ concentration increased, the sensor's voltage gradually decreased due to the reduction in the sensor's resistance, providing insights into self‐powered NH_3_ sensing (Figure 6e). Under a pressure of 11 N and a speed of 11 cm s^−1^, the voltage response of the ZnO/GO‐based sensor was recorded during alternating exposures to 0 and 50 ppm NH_3_ (Figure 6f). The sensor exhibited response and recovery times of 16 and 24 s, respectively, closely matching the measurements obtained with a multimeter, thereby validating the feasibility of the self‐powered sensing system. The reproducibility test of the ZnO/GO sensor powered by the WCS‐TENG demonstrated that this method had good consistency (Figure 6g).

## 3 Discussion

An ideal NH_3_ sensing material should exhibit specific adsorption toward NH_3_ gas molecules and possess an efficient signal amplification mechanism to ensure high selectivity and sensitivity. Fast and reversible adsorption of NH_3_ on the sensor surface is essential for achieving rapid response and recovery under practical operating conditions. In recent years, a variety of material systems and sensing mechanisms have been explored for NH_3_ detection. Polymer‐based materials have been widely employed to construct flexible or low‐temperature NH_3_ sensors owing to their excellent processability and strong interaction with NH_3_ molecules.^[^ ^39^, ^40^ ^]^ For example, NH_3_ sensors fabricated using polyaniline (PANI) nanofilms demonstrated good linear response with detection limits down to 10 ppm.^[^ ^41^ ^]^ However, conductive polymer sensors generally suffer from poor environmental stability and significant signal drift, which limit their applicability in complex environments.^[^ ^42^ ^]^ In contrast, metal oxides have emerged as promising gas sensing materials due to their high stability, structural diversity, and favorable electronic transport properties. Gas sensors based on materials such as WO_3_, SnO_2_, and In_2_O_3_ have been reported to exhibit high sensitivity and excellent humidity resistance.^[^ ^43^, ^44^, ^45^ ^]^ Furthermore, research efforts focus on developing advanced, multifunctional materials that not only improve sensitivity, selectivity, and long‐term stability but also integrate additional functionalities such as energy storage for self‐powered sensing applications.

Metal oxides, especially transition metal oxides, such as WO_3_, PdO, and PtO_2_, have become promising NH_3_ sensing materials due to the high stability, structural diversity, and favorable electronic transport properties.^[^ ^43^, ^44^, ^45^, ^46^, ^47^, ^48^, ^49^, ^50^, ^51^, ^52^ ^]^ In this work, we established a one‐step in situ polymerization method to synthesize low‐cost zinc‐doped carbon nanocomposites, i.e., ZnO/GO nanocomposites, used for the fabrication of supercapacitors and gas sensors simultaneously. The ZnO/GO‐based supercapacitor achieved a specific capacitance of 131 F g^−1^ at the current density of 1 A g^−1^, with the capacitance retention of 94% after 100 000 charge‐discharge cycles. Simultaneously, the sensor achieved high sensitivity detection within the 0.1–50 ppm range at room temperature, with the detection limit as low as 0.1 ppm and response/recovery times of 17 and 26 s (@10 ppm), respectively. It also exhibited excellent selectivity against interfering gases such as acetone and ethylamine. DFT calculations revealed that the composite material exhibited a notably higher NH_3_ adsorption energy (−1.878 eV) than the individual ones. The orbital hybridization between Zn 3d and C/O 2p orbitals was identified as the key mechanism enhancing gas selectivity and sensing performance. As detailed in Table S3 (Supporting Information), detection limits, response, and response/recovery times were listed with previously reported NH_3_ gas sensors (PANI, SrGe_4_O_9_/PANI, PPyNWs, MCNT, MCNT/PPy, TfmpoPcCo/MCNT, Bi_2_S_3_, PPy‐GO‐WO_3_, NiO/PANI, α‐Fe_2_O_3_/graphene, In_2_O_3_/Co_3_O_4_, Pt/ZnO/g‐C_3_N_4_, PFOTES‐Ti_3_C_2_T_x_‐CNF, BN‐H/P‐BNT, DM‐PG, MXene/CuO, AgNW/p‐SiNM, ZnO‐SnO_2_, Pt/N‐mWO_3_, and ZnO/GO).^[^ ^46^, ^47^, ^48^, ^49^, ^50^, ^51^, ^52^, ^53^, ^54^, ^55^, ^56^, ^57^, ^58^, ^59^, ^60^, ^61^ ^]^ The ZnO/GO‐based sensor highlighted its comprehensive advantages over other counterparts in working temperature, sensitivity, and detection limit. Impressively, it demonstrated outstanding response and recovery times among them under exposure to 10 ppm NH_3_. These results suggested that the ZnO/GO‐based NH_3_ gas sensor exhibited outstanding sensing characteristics, thus being an ideal candidate for NH_3_ monitoring.

Mechanistically, pure *n*‐type ZnO forms chemisorbed oxygen species (e.g., O_2_^−^, O^−^, O^2−^) on its surface in air, generating an electron depletion layer that increases resistance.^[^ ^37^ ^]^ Upon exposure to the reducing gas NH_3_, electrons from NH_3_ were returned to the ZnO conduction band via surface redox reactions, while reaction products (e.g., N_2_ and H_2_O) were released, leading to a thinner depletion layer, an increased carrier concentration, and a decrease in resistance (Figure 1f).^[^ ^3^, ^43^, ^44^, ^45^ ^]^ GO, with abundant oxygen‐containing functional groups (e.g., epoxy, hydroxyl, carboxyl) on its sheets, exhibited *p*‐type semiconductor behavior, with conductivity dominated by holes.^[^ ^27^, ^30^ ^]^ In air, adsorbed oxygen stabilized the hole concentration, while NH_3_ donated electrons that recombined with holes, decreasing carrier concentration and increasing resistance (Figure S13, Supporting Information).^[^ ^38^ ^]^ In the ZnO/GO nanocomposites, GO not only provided intrinsic *p*‐type behavior but also offered a large surface area and abundant adsorption sites for NH_3_. Acting as a “sponge,” GO efficiently captured and concentrated NH_3_ molecules and transported them to adjacent ZnO nanoparticles, enhancing the reaction efficiency between NH_3_ and ZnO. This synergy significantly improved the sensing and energy storage performance.^[^ ^38^ ^]^ Moreover, the response of this sensor to ethylamine (C_2_H_5_NH_2_, 45.8%) was higher than that to other interfering gases such as acetone and nitrogen dioxide (29.66%–23.17%), although still lower than that to NH_3_ (68.2%) (Figure 5a). We attributed this phenomenon to the comparable basicity and electron‐donating capability between C_2_H_5_NH_2_ and NH_3_.^[^ ^3^, ^62^ ^]^ In practical environmental monitoring, where the concentration of ammonia was relatively low while other interfering gases, especially ethylamine, were present at higher levels, it was recommended to employ a multi‐sensor array combined with analytical algorithms to achieve accurate multi‐component detection.^[^ ^43^ ^]^

**Figure 7:**
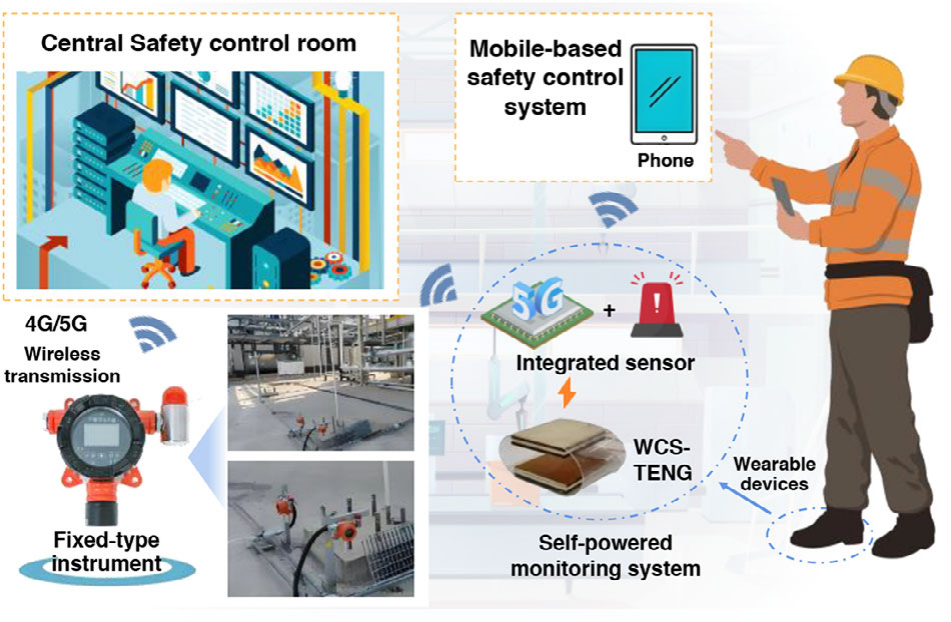
Schematic diagram of application scenarios for fixed‐type and self‐powered ammonia monitoring systems.

Two application scenarios, including an ammonia gas detector designed for fixed‐point monitoring and a portable system integrated with a footwear‐incorporated wearable TENG, in which the biomechanical energy generated during walking was harnessed for self‐powered ammonia sensing, were verified (**Figure** **7**). The fixed‐type detector enabled continuous monitoring of NH_3_ concentrations at a designated site and transmitted the data to a computer via a wireless connection, while the portable counterpart allowed inspection personnel to perform real‐time on‐site monitoring. To minimize the influence of temperature and humidity on metal oxide‐based gas sensors, a common approach in previous studies was to perform numerical calibration using temperature and humidity dependence curves in combination with real‐time data from separate temperature/humidity sensors.^[^ ^39^, ^40^, ^43^ ^]^ In contrast, we adopted the instrument packaging structure as shown in Figure 4 to mitigate the effect of humidity on the sensor device. During stability and environmental endurance testing, excellent repeatability and reproducibility were confirmed through consistent response–recovery behavior and negligible signal variation among multiple sensors during long‐term operation. The response relative standard deviation remained below 1% over 210 days of continuous testing, demonstrating outstanding durability and reliability. Field evaluation further validated the sensor's stability, as it continuously recorded NH_3_ concentrations of ≈5–6 ppm near an industrial pipeline over six months, consistent with actual environmental conditions. For the portable system, the WCS‐TENG was developed to harvest mechanical energy from a contact‐separation setup that mimicked human footsteps, achieving a maximum output power of 4.1 mW. Notably, in the majority of existing studies, the alternating voltage generated by wearable TENGs was first rectified or stored to obtain a direct current output suitable for powering electronic devices.^[^ ^8^, ^10^, ^23^ ^]^ In contrast, the ZnO/GO sensor developed in this work featured low power consumption and exhibited a well‐defined resistive sensing behavior, and its operating voltage range was inherently aligned with the output characteristics of the TENG. As a result, the sensor was able to be directly powered by the TENG without the need for additional rectification or voltage regulation circuits, highlighting the technological advantage of achieving self‐powered operation in this portable system. Compared to the fixed‐point detector, this self‐powered design allowed continuous monitoring and on‐site alerting without an external power supply, enhancing practicality and reliability in dynamic environments. These two innovative applications not only advanced the intelligent and networked development of NH_3_ monitoring systems but also significantly enhanced the spatial coverage and operational flexibility of NH_3_ concentration monitoring.

## 4 Conclusion

In this study, the ZnO/GO nanocomposites were successfully fabricated via an in situ polymerization method, exhibiting a unique structure with uniformly distributed ZnO nanoparticles on GO sheets. This architecture endowed the materials with dual functionalities: as supercapacitor electrodes, they delivered a high specific capacitance of 131 F·g^−1^ at 1 A·g^−1^ and outstanding cycling stability (94% retention over 100 000 cycles); as ammonia gas sensors, they achieved high sensitivity across 0.1–50 ppm, fast response/recovery times of 17/26 s (10 ppm), and excellent selectivity against interfering gases. Long‐term fixed‐point monitoring demonstrated remarkable stability, with a response relative standard deviation below 1% over 210 days, consistent with actual environmental conditions. Furthermore, the integration of a wearable contact‐separated TENG enabled self‐powered operation of the ZnO/GO‐based ammonia sensor, supporting continuous NH_3_ detection in dynamic environments without external energy input. The multi‐scenario applications enhanced the spatial coverage and operational flexibility of NH_3_ concentration monitoring, providing a promising pathway toward intelligent and sustainable environmental sensing technologies.

## 5 Experimental Section

### Materials

Zinc nitrate hexahydrate [Zn(NO_3_)·6H_2_O] (99 wt.%, 300 mesh), 2‐methylimidazole [C_4_H_6_N_2_] (99.5 wt.%, 500 mesh), hydrochloric acid [HCl] (37 wt.%), and sulfuric acid [H_2_SO_4_] (98 wt.%) were provided by China National Pharmaceutical Group Chemical Reagents Co., Ltd. (Shanghai, China). Aluminum powder (99.6 wt.%, 200 mesh) was supplied by Xingrongyuan Company (Beijing, China). Graphene oxide [GO] (99 wt.%, 200 mesh) was supplied by Chengdu Organic Chemical Company (Chengdu, China). The current collector and activated carbon electrode of the button‐type supercapacitor were provided by Taiwan Zhifengwei Technology Co., Ltd. Polyvinyl alcohol (PVA) (99 wt.%, 400 mesh) was provided by Keleli International Trading Shanghai Co., Ltd. The dry gas samples for gas sensor calibration were supplied by Dalian Special Gas Co. Ltd (China). All solution was prepared by deionized (DI) water (18.2 MΩ cm).

ZnO/GO nanocomposites were prepared via the in situ polymerization method (Figure S1, Supporting Information). First, 10 mmol of Zn(NO_3_)_2_·6H_2_O and 15 mmol of C_4_H_6_N_2_ (2‐MIM) were dissolved and thoroughly mixed in 500 mL of DI water. The mixture was stirred at 35 °C for 4–6 h to facilitate the formation of ZIF‐8. The formed ZIF‐8 was placed in a muffle furnace and heated to 400 °C at a rate of 5 °C·min^−1^, then maintained at 400 °C for 2 h. After naturally cooling to room temperature, ZIF‐8‐derived ZnO (hereafter referred to as ZnO) was obtained in the form of spherical nanostructure ≈100 nm in size (Figure S2a, Supporting Information). In the next step, 10 mmol of the formed ZnO, 5 mmol of GO, and 5 mmol of Al powder were mixed. Under slow stirring, the mixture was gradually added to 500 mL of 1 m (mol L^−1^) HCl solution. The HCl solution was prepared by diluting concentrated HCl with DI water. The reaction happened under acidic conditions, where Al reacted with H⁺ ions, releasing heat and reducing the epoxy group (─C─O─C) of GO to phenol hydroxyl groups (─C─OH). This resulted in the formation of a composite material consisting of edge‐oxygen‐rich graphene and ZnO, with 1,5‐dihydroxyanthraquinone and 2,6‐diaminoanthraquinone molecules confined between the graphene sheets and ZnO. The entire composite formation process lasted ≈2–3 h, and the reaction was carried out at room temperature (≈25 °C) with vigorous stirring to ensure uniform mixing.

### Fabrication Process—Fabrication of Supercapacitor

The button‐type supercapacitor consisted of a current collector, a self‐fabricated ZnO/GO composite material, a separator, an activated carbon electrode, and an electrolyte. The H_2_SO_4_/PVA gel electrolyte was prepared by mixing 2.1 g of PVA with 90 mL of DI water at 75 °C. Afterward, 8 g of H_2_SO_4_ was added to the solution while stirring. The H_2_SO_4_/PVA gel electrolyte naturally evaporated and served as both the electrolyte and the separator.

### Fabrication Process—Fabrication of ZnO/GO Gas Sensor

A smooth‐surfaced, flexible, and mechanically strong polyethylene terephthalate (PET) substrate was selected as the base material for the sensor. The flexible interdigital electrodes were fabricated on the PET substrate using a micro‐electromechanical systems (MEMS) process. The fabrication steps included magnetron sputtering, copper plating, photolithography, etching, and gold plating. First, a 12 µm thick copper forked electrode was deposited on the PET substrate, and then 1 µm thick gold was deposited on the copper surface to enhance conductivity. The sensor dimensions were ≈1 cm × 1 cm. Both the line spacing and the width of the interdigital electrodes were 75 µm in depth.

The NH_3_ sensing film was prepared by spin‐coating. A dispersion liquid was obtained by sonicating 1 g of ZnO, GO, or ZnO/GO sensing material in 20 g of DI water for 20 min. The interdigital electrodes were fixed at the center of the base using double‐sided tape, and 100 mg of dispersion liquid was drop‐cast onto the electrode surface. The spin‐coating instrument (WS‐650MZ‐8NPPB, Laurell Technologies, USA) was then activated with a rotational speed of 800 rpm to form a uniform coating on the electrode surface due to centrifugal force. Finally, the coating was dried to obtain a uniformly adhered thin film.

### Characterization

The SEM (Merlin Compact), TEM (JEM‐2100F), XRD (D8 Advance), XPS (EscaLab 250Xi), Raman (LabRAM HR Evolution), and Brunauer–Emmett–Teller (BET) analyzer (ASAP‐2020‐Plus) were used to characterize the system. The electrochemical workstation (CHI 660E) was used to measure the supercapacitor performance. The Agilent Data Acquisition Instrument (Agilent 34970A) was used to record the resistance values of the fabricated sensor. The Keysight Electrometer (Keysight 6514) was used to record the electrical output of the manufactured WCS‐TENG.

### Statistical Analysis

In this work, no data was preprocessed. Figures 3d and 5g and Figures S11 and S14 (Supporting Information) used sample means with a sample size of 3, and error bars represent the standard deviation of three independent experiments. Figure 5f used sample means with a sample size of 5, and error bars represent the standard deviation of five independent experiments. Statistical analysis was performed using Origin 2021 software. The data in Figure 6c utilized the maximum values from the samples. All remaining data were presented as raw data.

## Conflict of Interest

The authors declare no conflict of interest.

## Author Contributions

X.W., L.G. contributed equally to this work. X.W. did conceptualization, methodology, data curation, and writing of the original draft. L.G. did the investigation, methodology, and writing of the review & editing. X.Z. did the writing of the review & editing, conceptualization, supervision, and funding acquisition.

## Supporting information



Supporting Information

Supporting Information

## Data Availability

The data that support the findings of this study are available from the corresponding author upon reasonable request.
