# Supplementary material for: Ammonia Gas Sensor Fabricated by Multifunctional ZnO/GO Nanocomposites for Long‐Term, Self‐Powered Monitoring
Source: Adv Sci (Weinh). 2025 Dec 5;13(10):e16833. doi: 10.1002/advs.202516833 (PMC12915130; doi:10.1002/advs.202516833)
Supplement: Supplementary file 1 — Supporting Information [file ADVS-13-e16833-s002.docx]

**Ammonia Gas Sensor Fabricated by Multifunctional ZnO/GO Nanocomposites for Long-term, Self-powered Monitoring**

Xingwei Wang^1^, Likun Gong^2^, Xiaohong Zhou^1†^

^1^ *State Key Laboratory of Regional Environment and Sustainability, School of Environment, School of Environment, Tsinghua University, Beijing 100084, China*

^2^ *College of Science, China University of Petroleum (East China), Qingdao, Shandong, 266580, China*

^†^*Corresponding authors:*

*xhzhou@mail.tsinghua.edu.cn (Xiaohong Zhou)*

**Fig. S1** Synthesis procedure and underlying formation mechanisms of ZnO/GO nanocomposites.


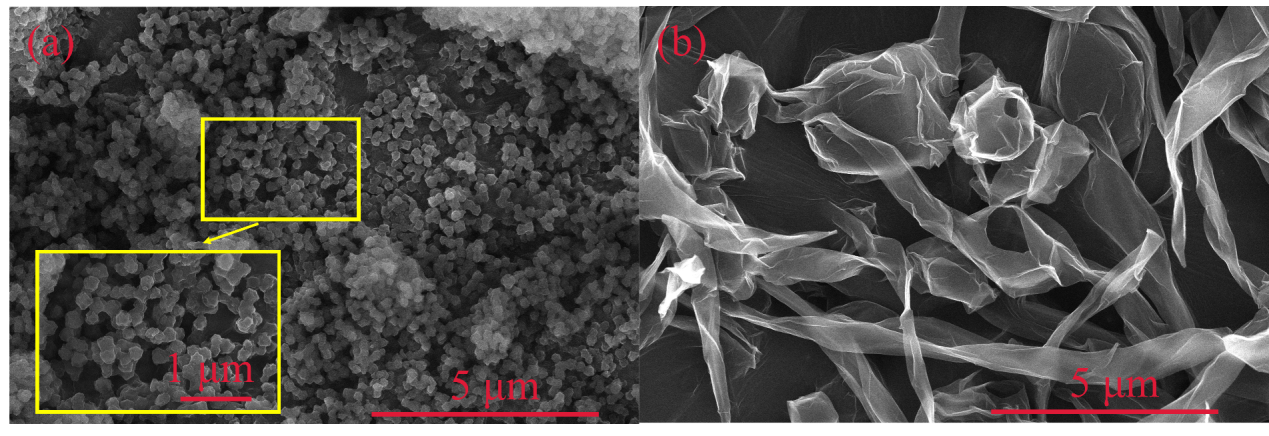


**Fig. S2** SEM images of (a) ZnO and (b) GO.

**
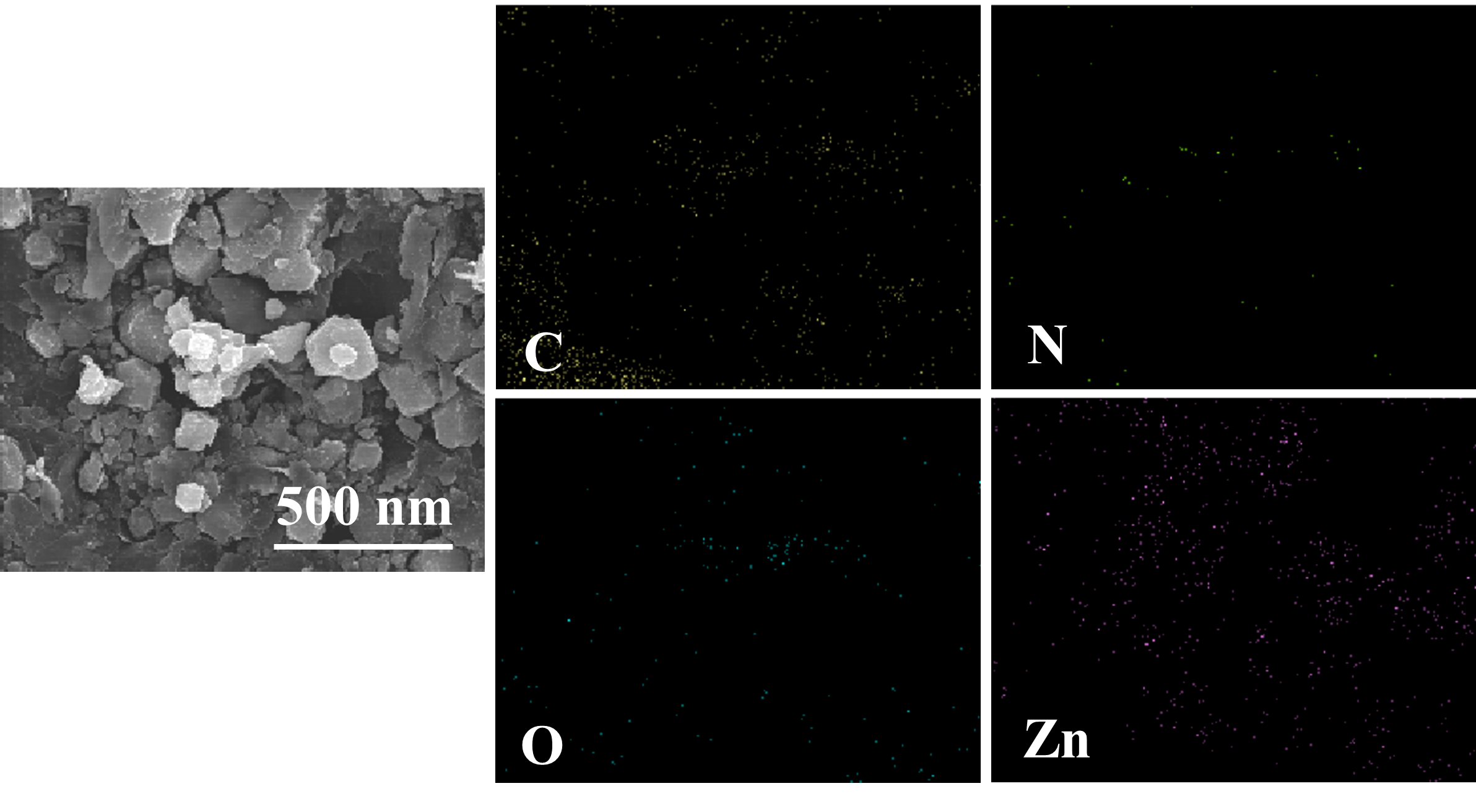
**

**Fig. S3** The SEM and elemental mapping images of ZnO/GO composite materials.


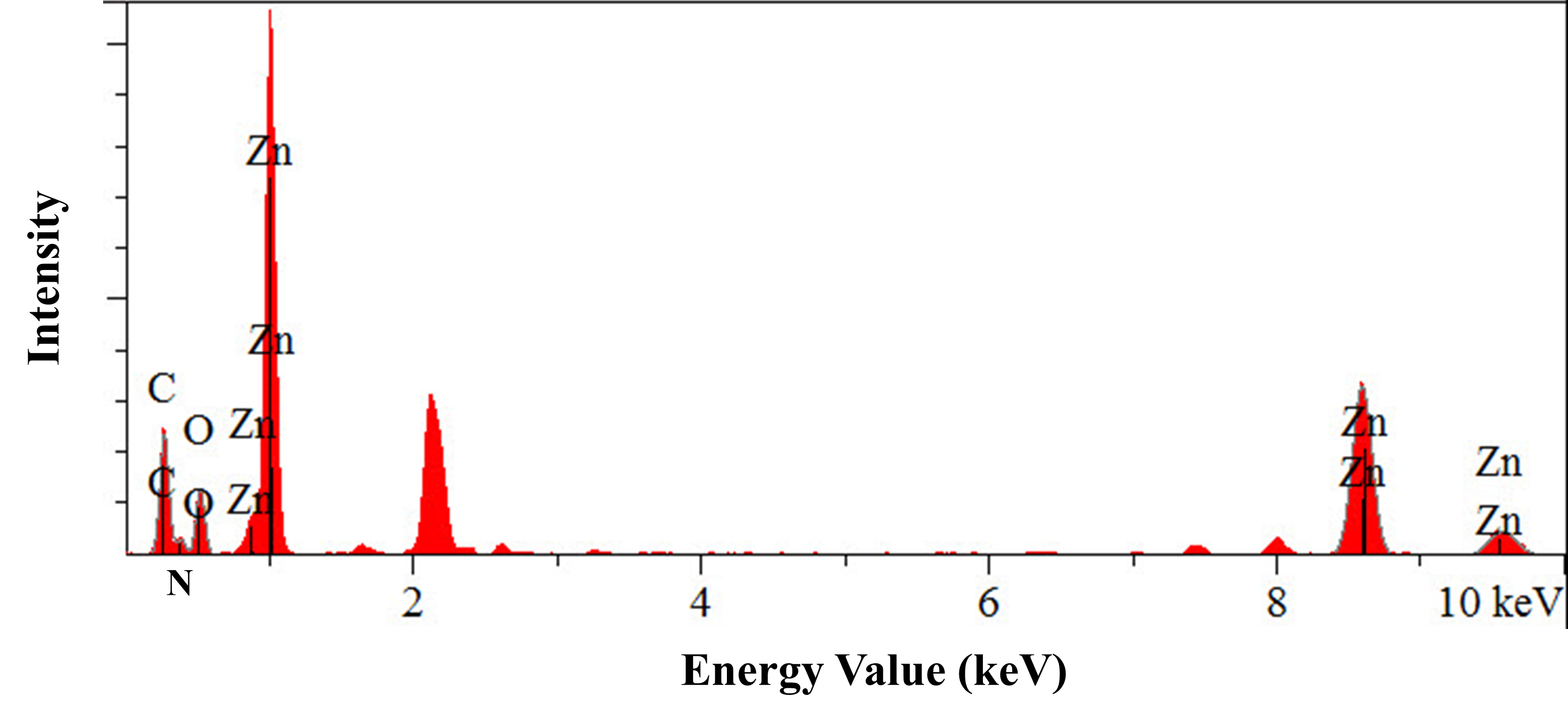


**Fig. S4** The EDS spectra of ZnO/GO composite materials.


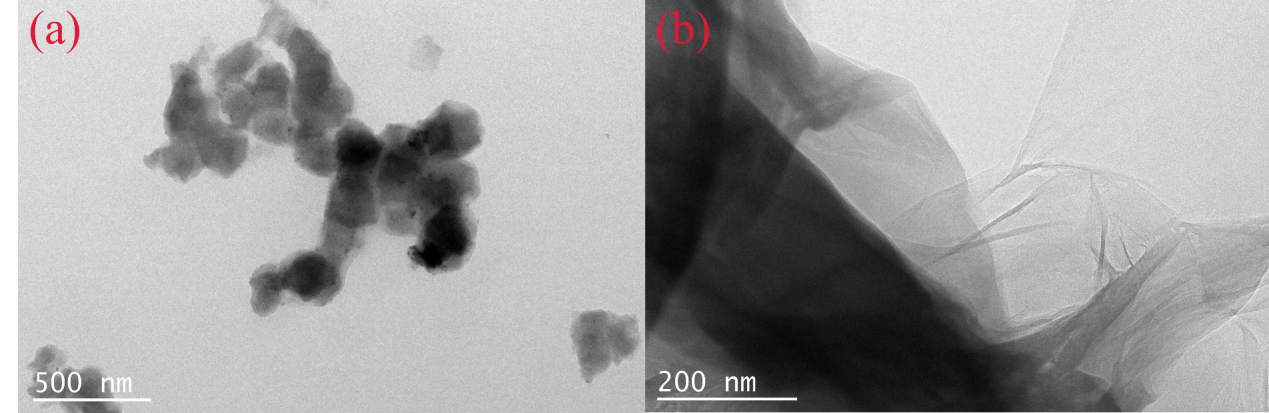


**Fig. S5** TEM images of (a) ZnO and (b) GO.


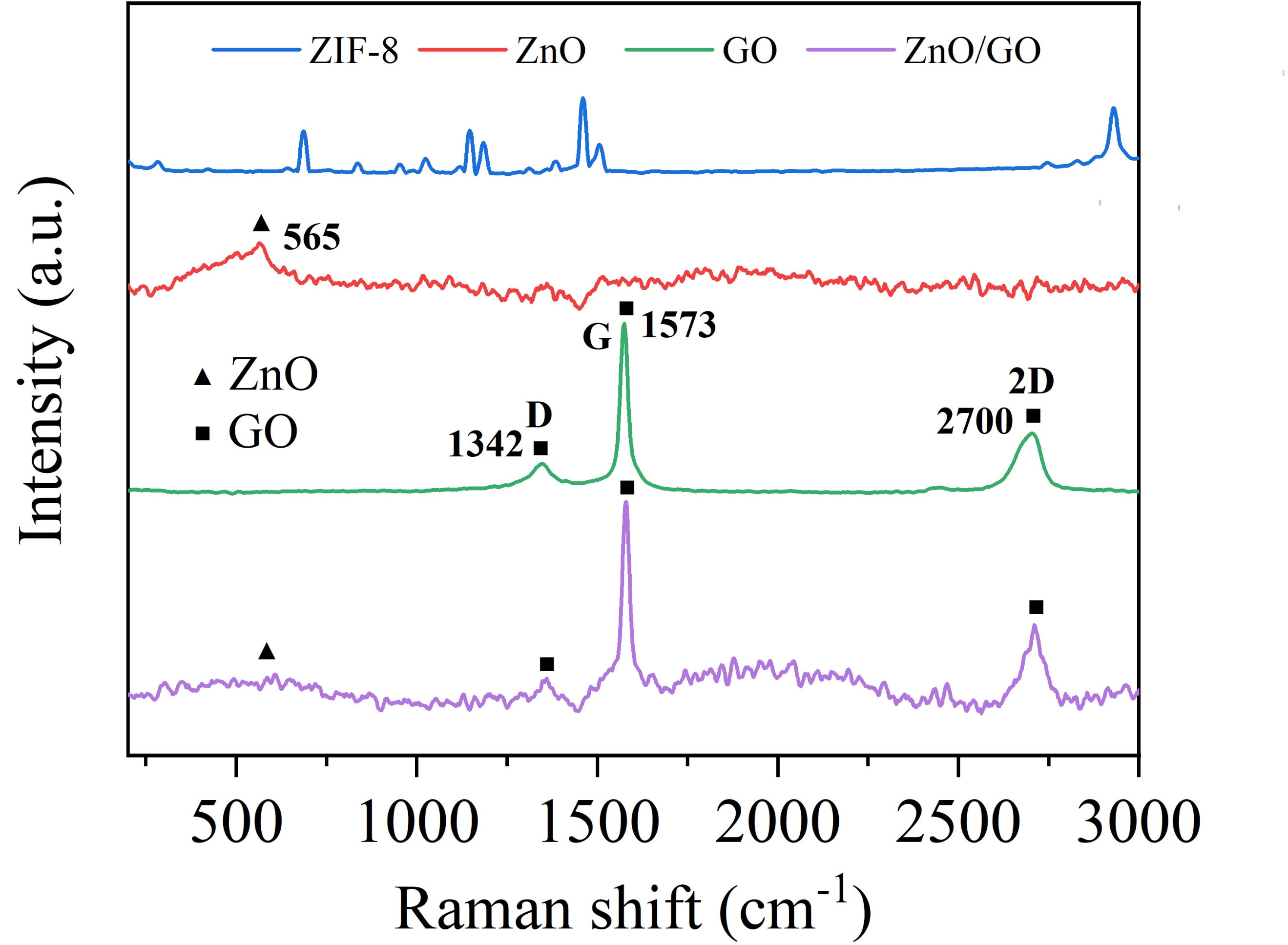


**Fig. S6** Raman spectra of ZIF-8, ZnO, GO, and ZnO/GO nanocomposite materials.

**
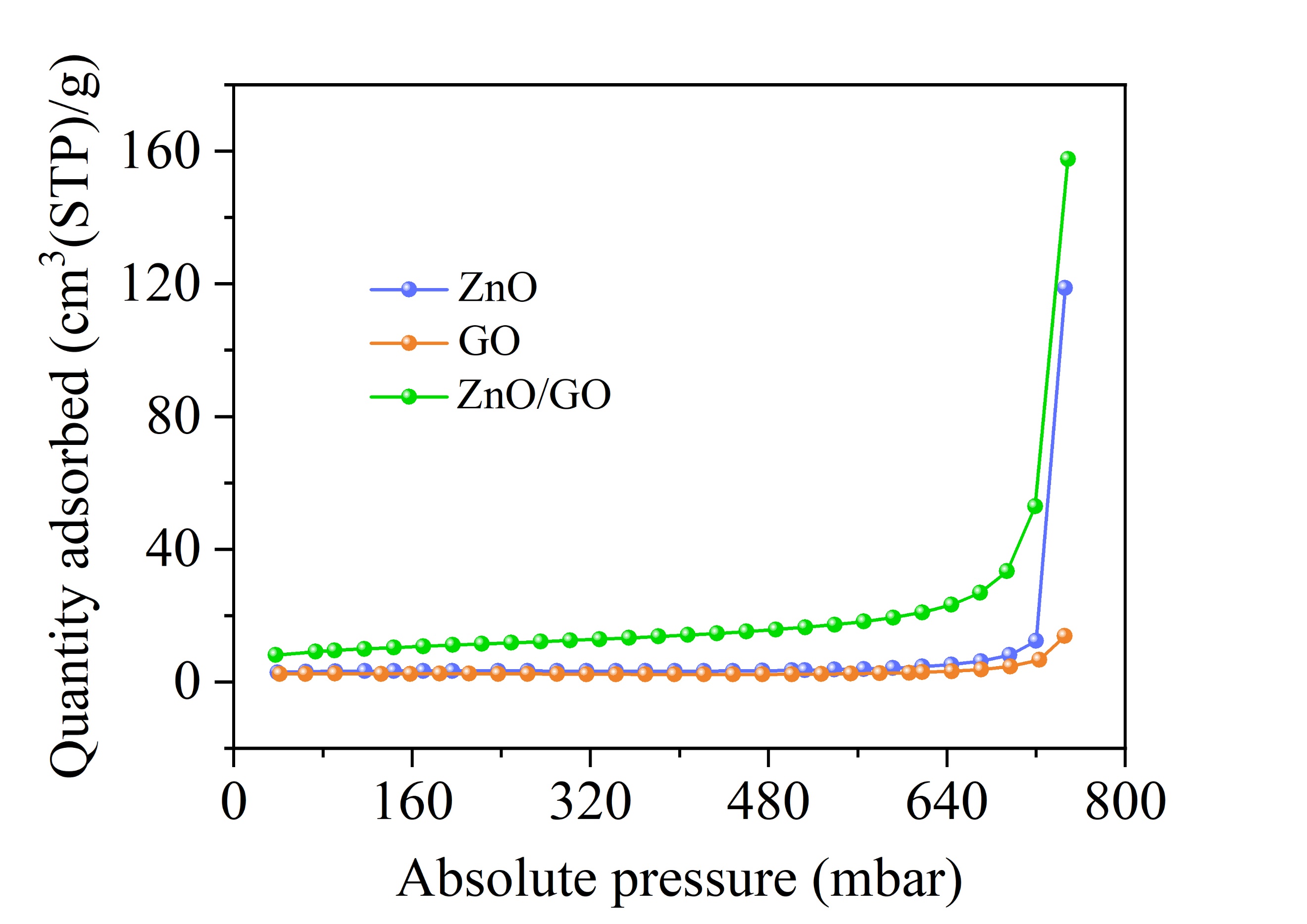
**

**Fig. S7** Nitrogen sorption isotherms measured at 77 K on the powder ZnO, GO and ZnO/GO.


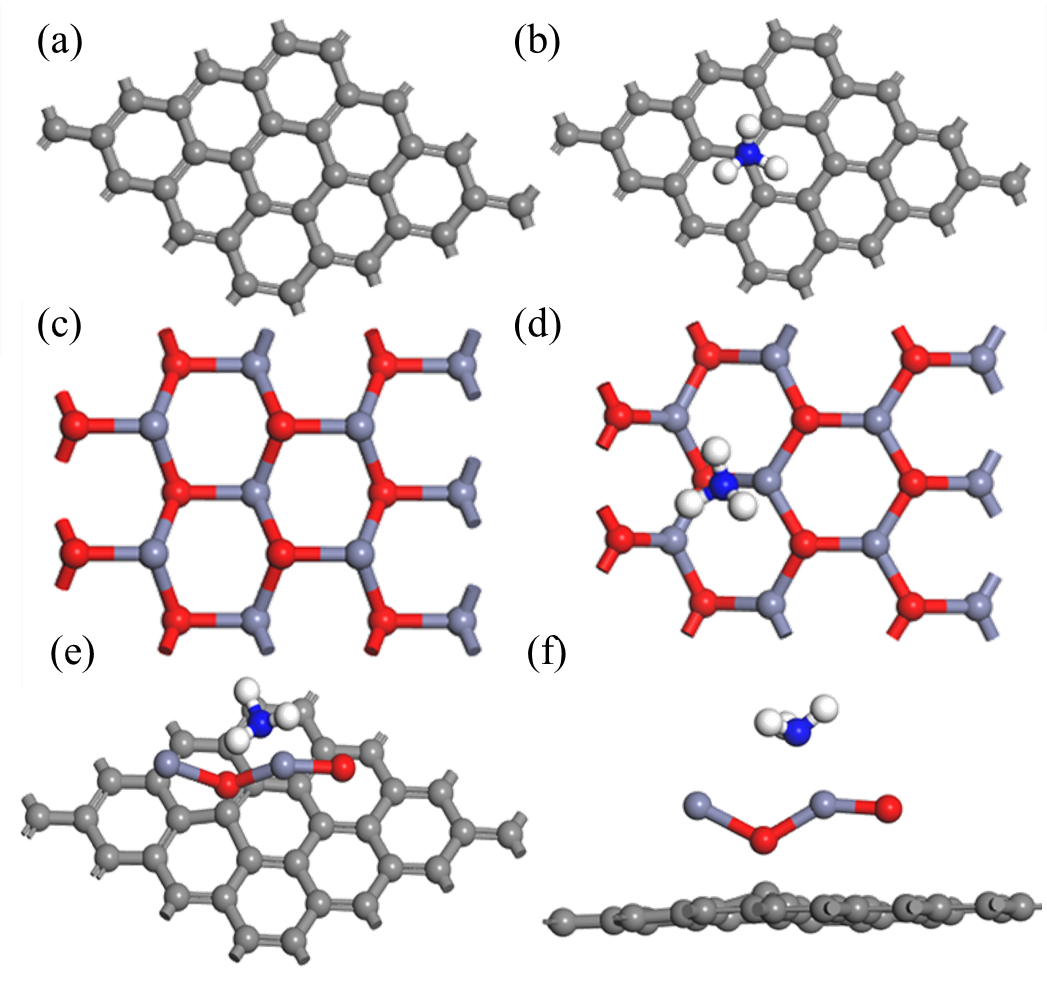


**Fig. S8** Model diagram used for first principles calculations. (a-b) Schematic diagrams of molecular models of GO surface before and after adsorption of NH_3_ molecules. (c-d) Schematic diagrams of molecular models of the ZnO surface before and after the adsorption of NH_3_ molecules. (e-f) Schematic diagram of molecular model of ZnO/GO composite structure after adsorption of NH_3_ gas.

**
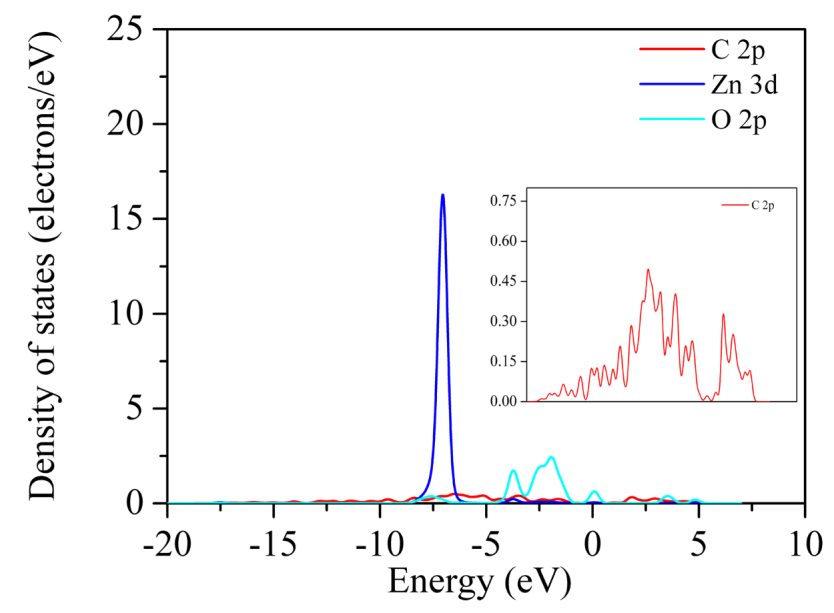
**

**Fig. S9** Projected density of states diagrams of ZnO/GO system before adsorption of NH_3_ molecules. Inset shows detailed data of C 2p in ZnO/GO.

**Fig. S10** Ragone plot of the ZnO/GO-based supercapacitor under different testing conditions.


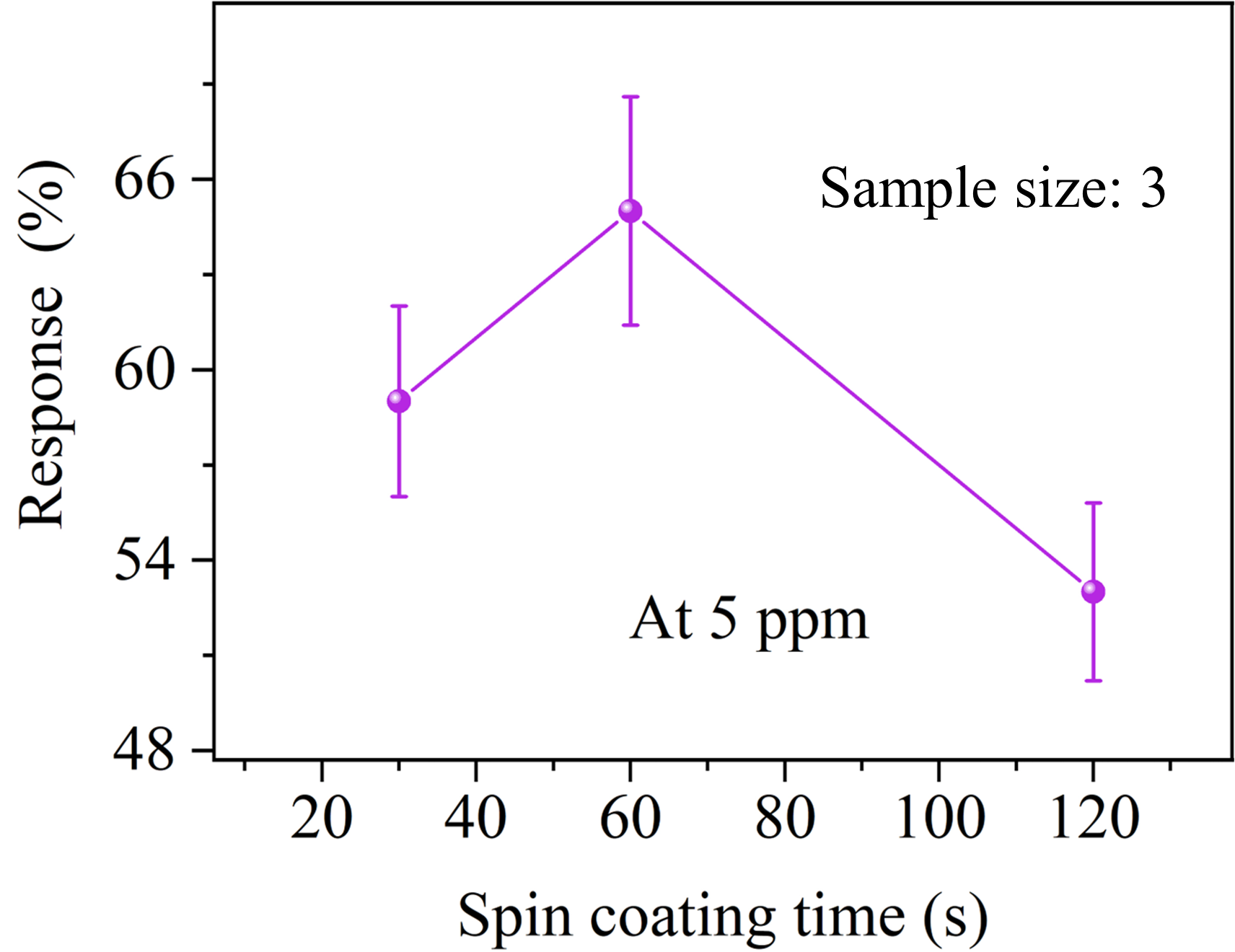


**Fig. S11** Effect of spin-coating time on the response of ammonia gas sensor.

**
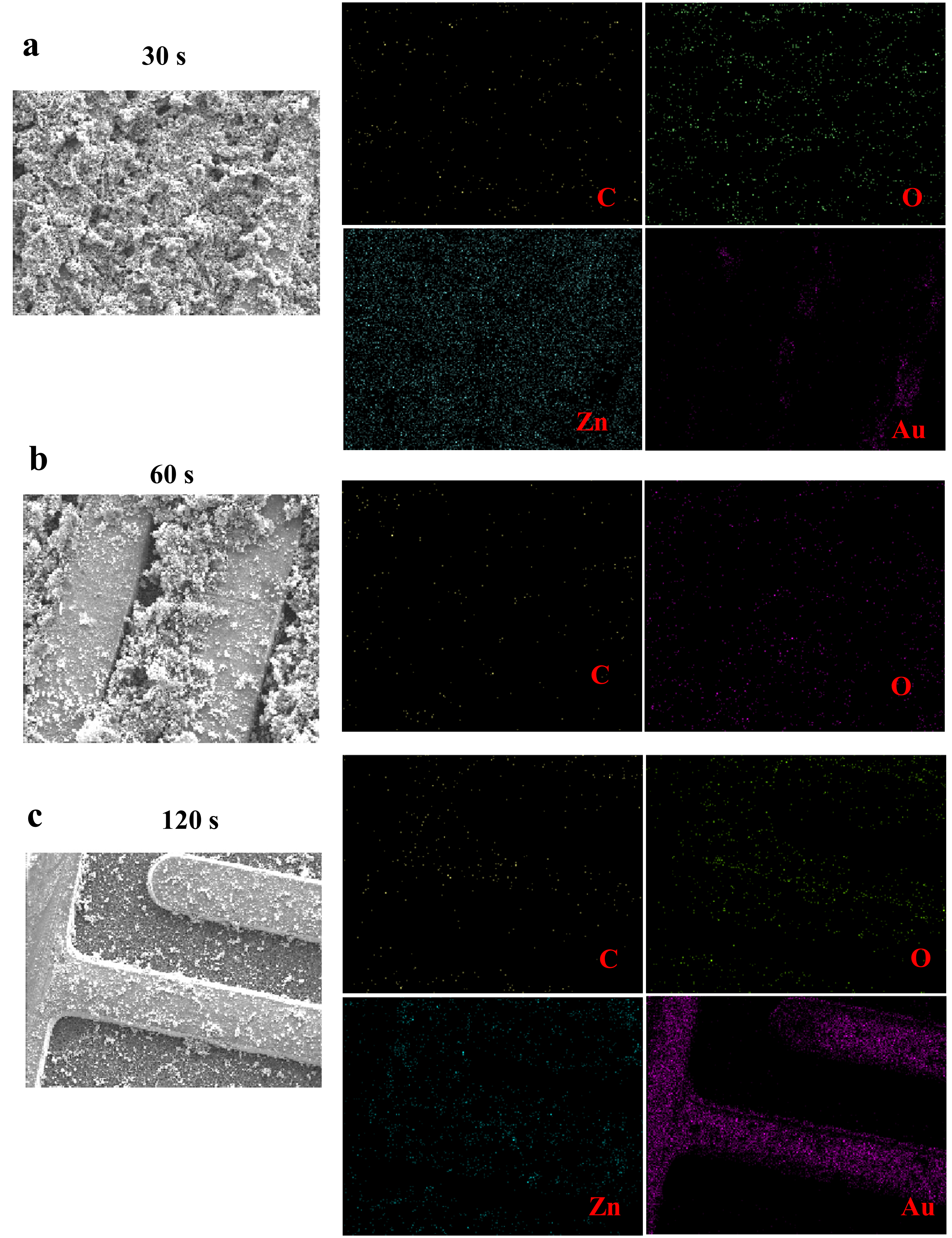
**

**Fig. S12** SEM images and elemental mapping of the sensor surface which were spin-coated with ZnO/GO nanocomposites for (a) 30 s, (b) 60 s, and (c) 120 s.


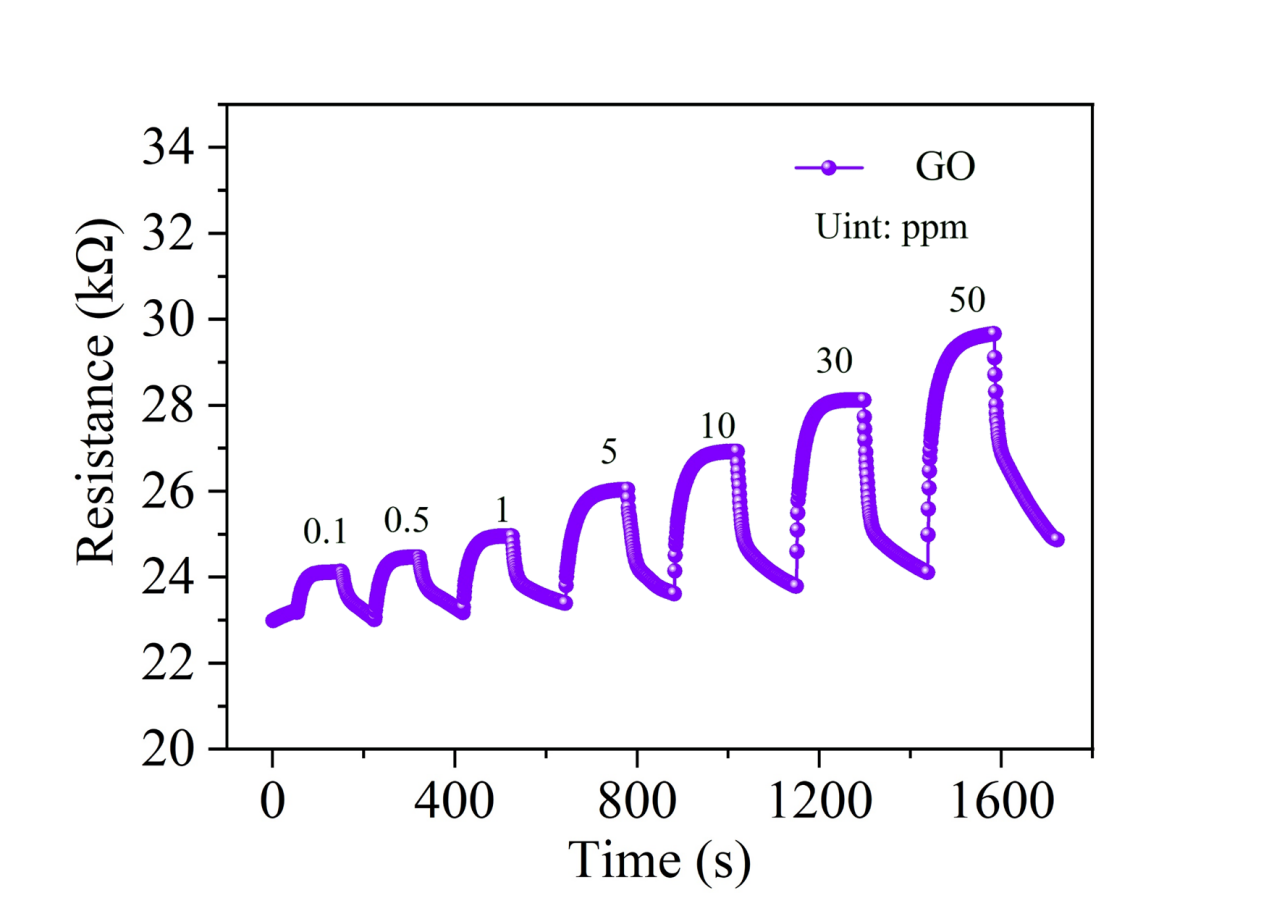


**Fig. S13** The resistance changes of gas sensors based on pure GO under different NH_3_ concentrations.


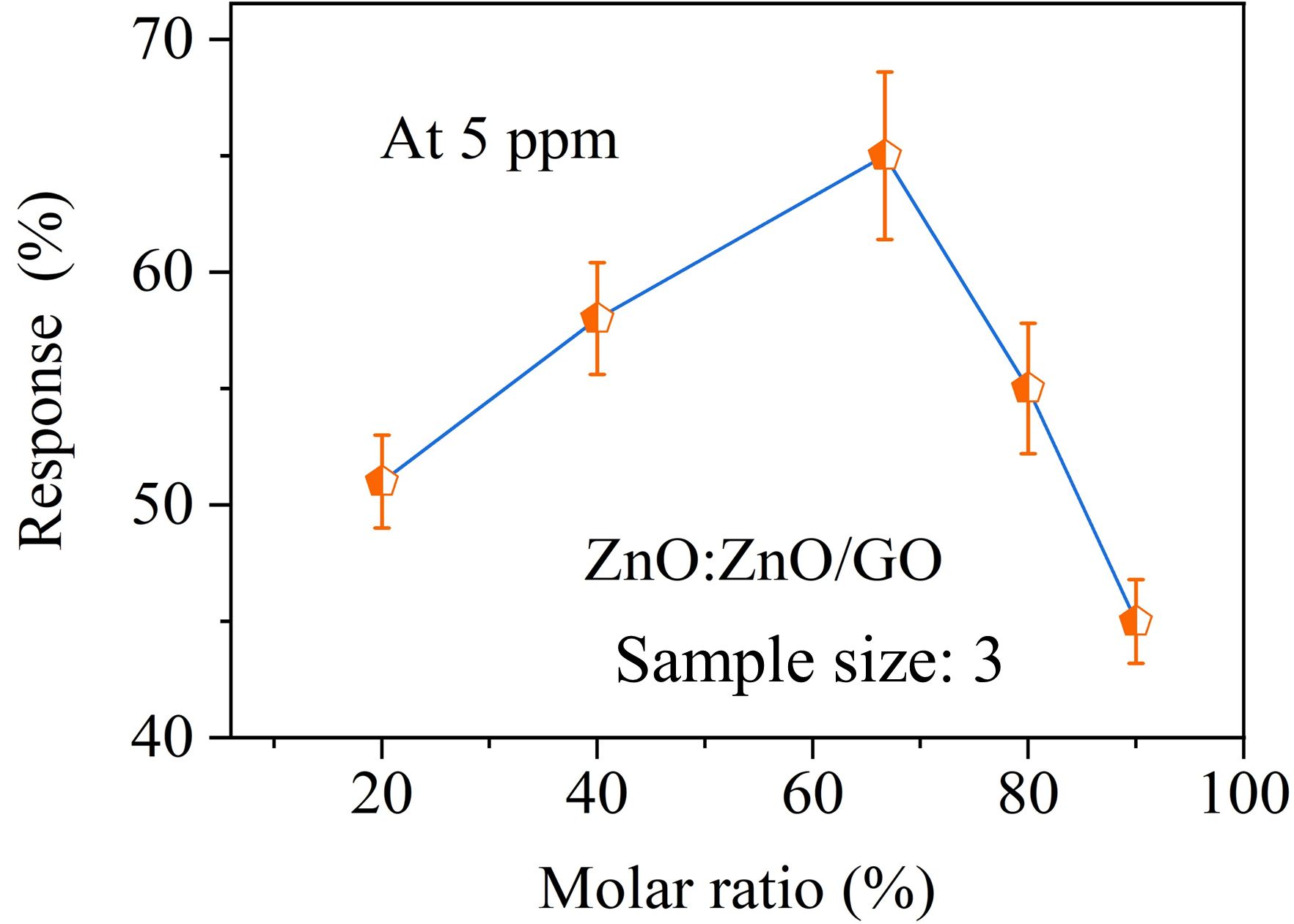


**Fig. S14** Effect of the molar ratio of ZnO in ZnO/GO nanocomposites on the response of ammonia gas sensor.


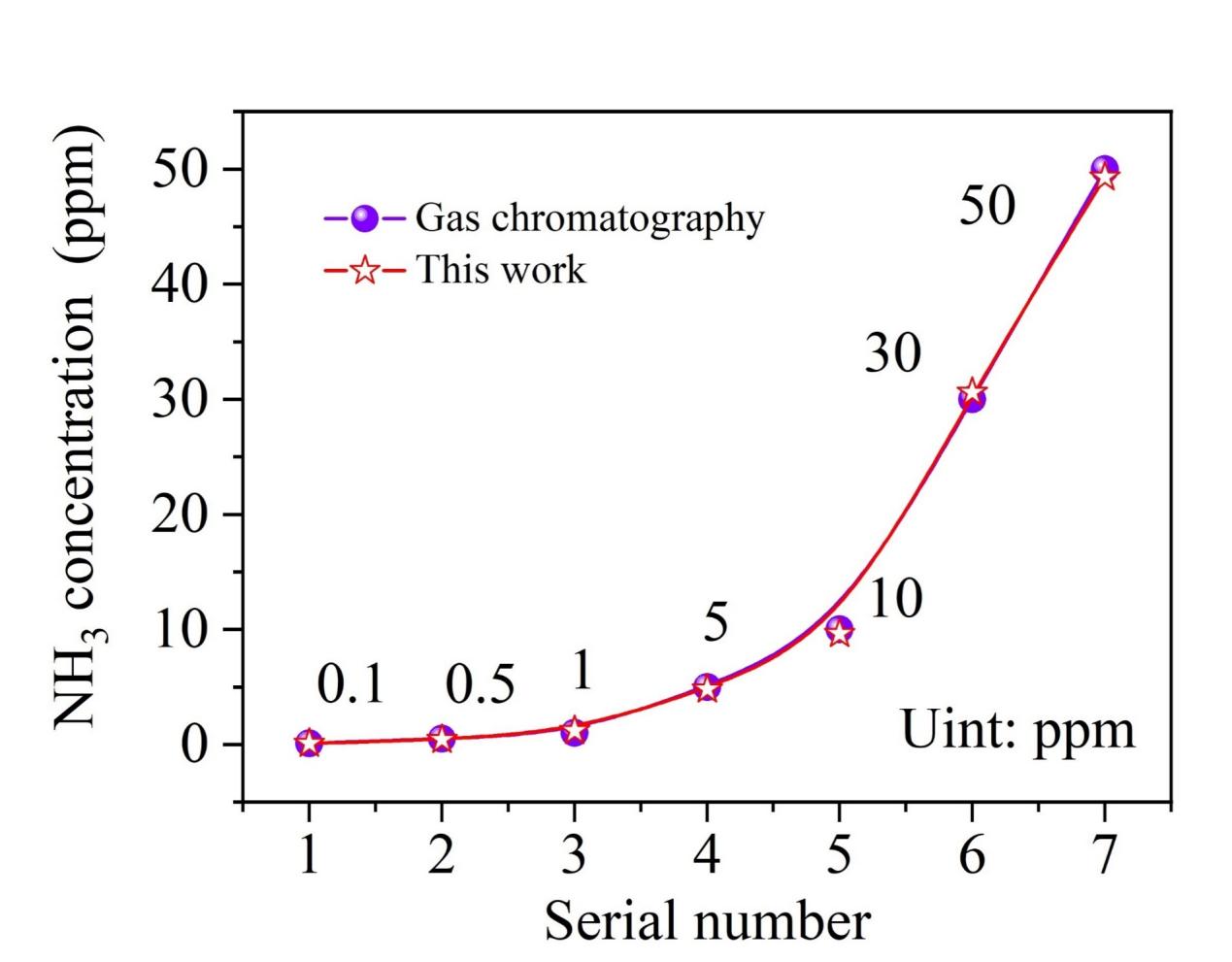


**Fig. S15** Results comparison between gas chromatography and NH_3_ concentration tested in this work.

**Fig. S16** Photographs of the circuit modules of the ammonia detector before (left) and after (right) assembly.

**
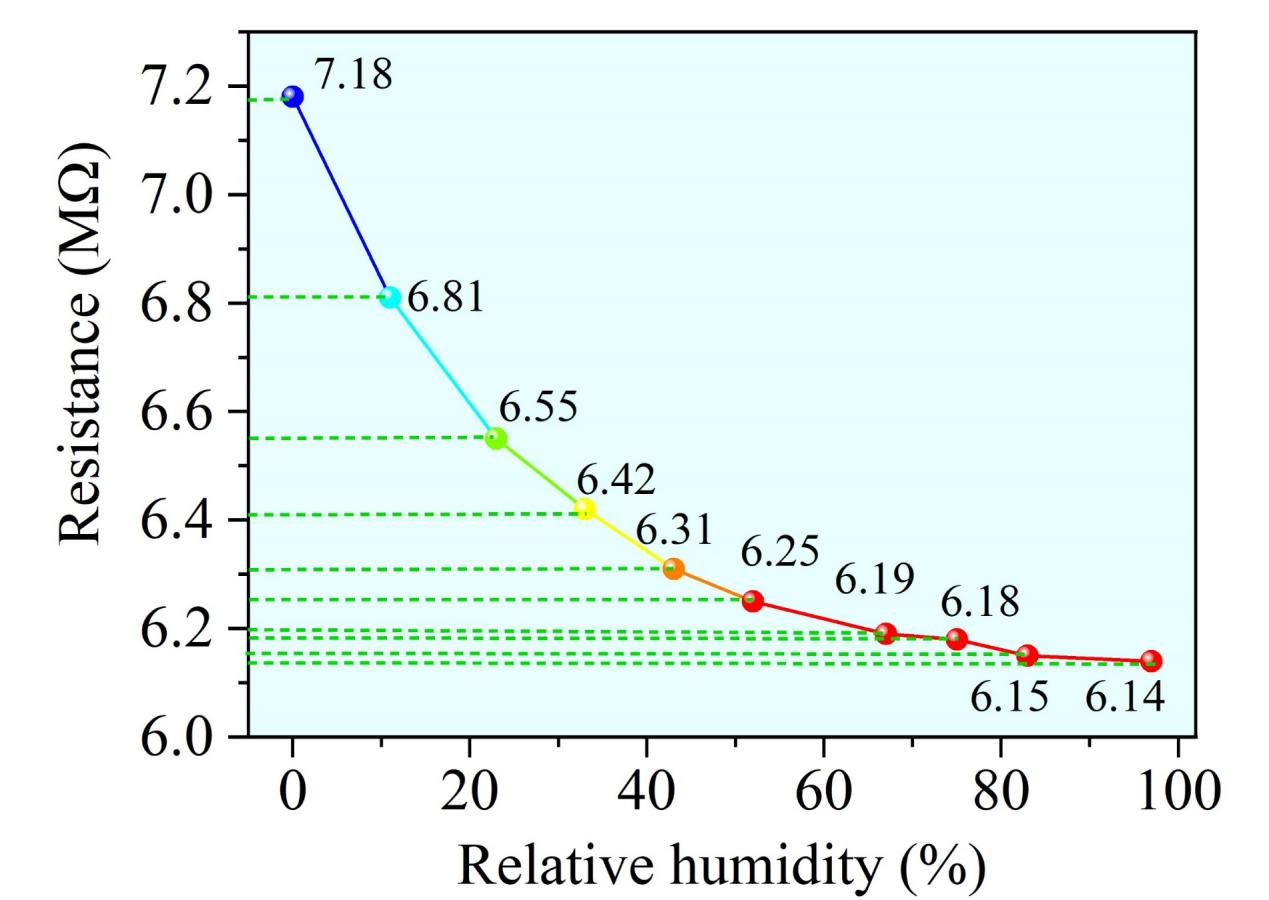
**

**Fig. S17** The effect of humidity on the base resistance of the ZnO/GO nanocomposites-based ammonia gas sensor.

**Fig. S18** The 60-day stability of five ZnO/GO sensors under exposure to 5 ppm ammonia concentration.

**
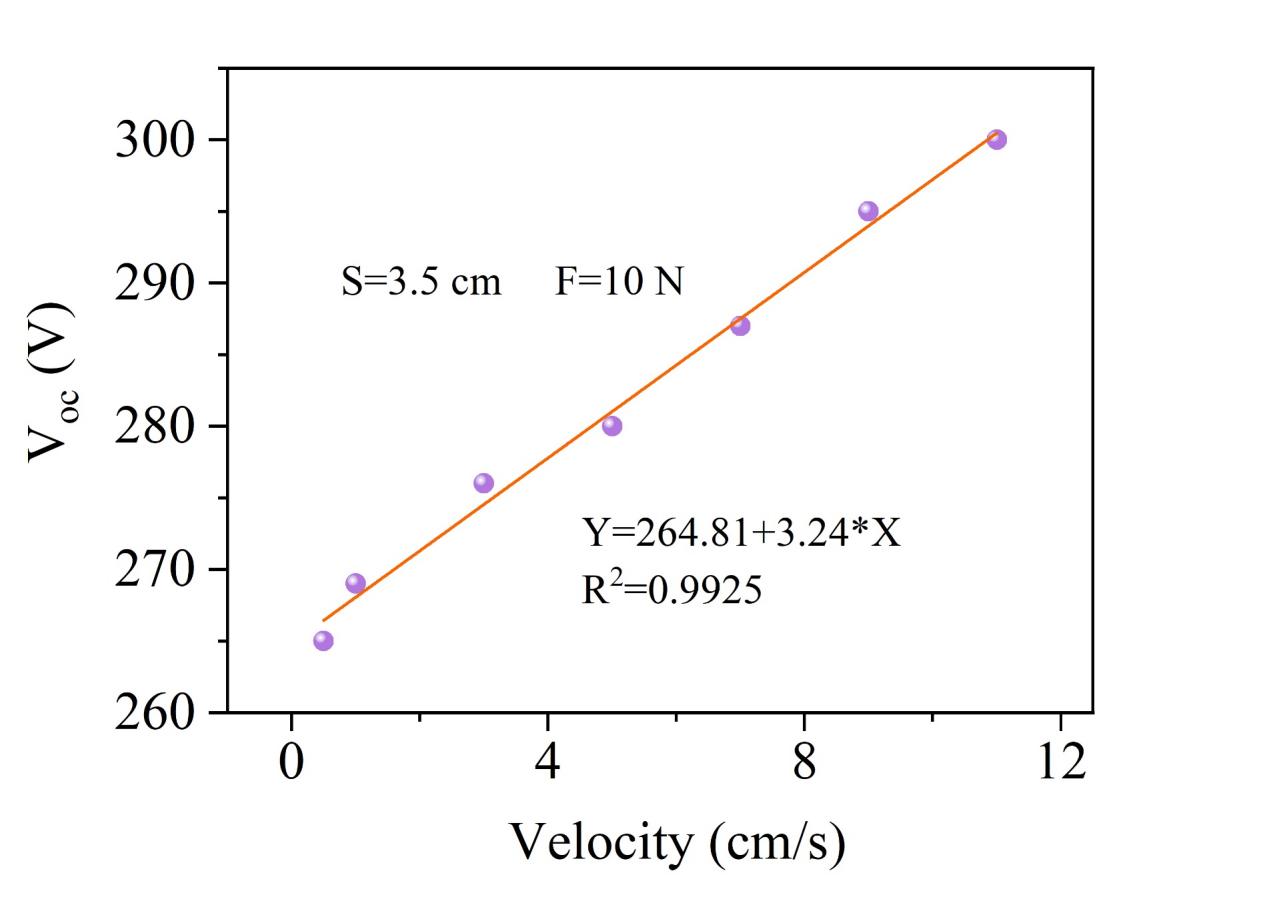
**

**Fig. S19** The fitting curve of velocity versus the open-circuit voltage of the WCS-TENG.

**
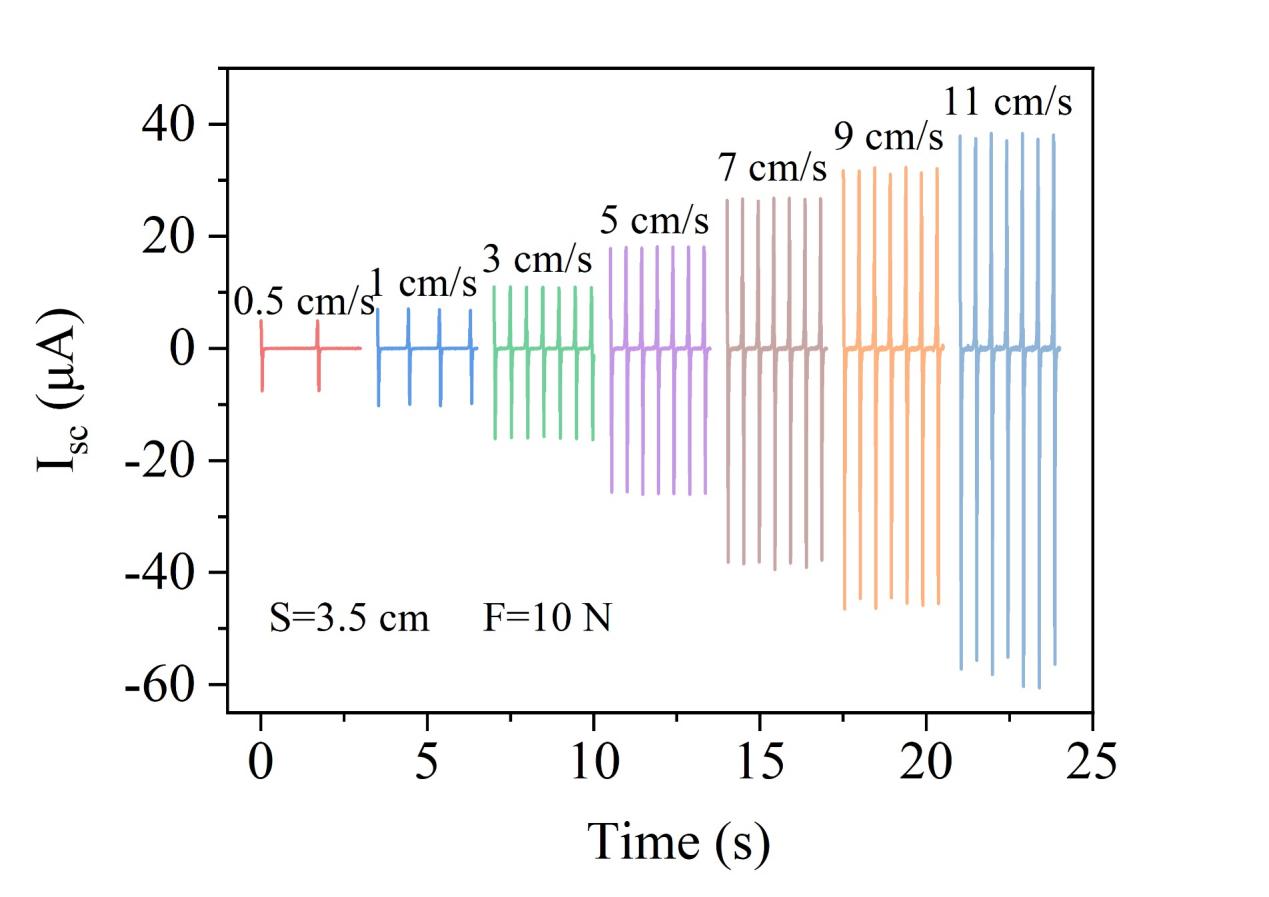
**

**Fig. S20** Effect of velocity on the short-circuit current of the WCS-TENG.

**
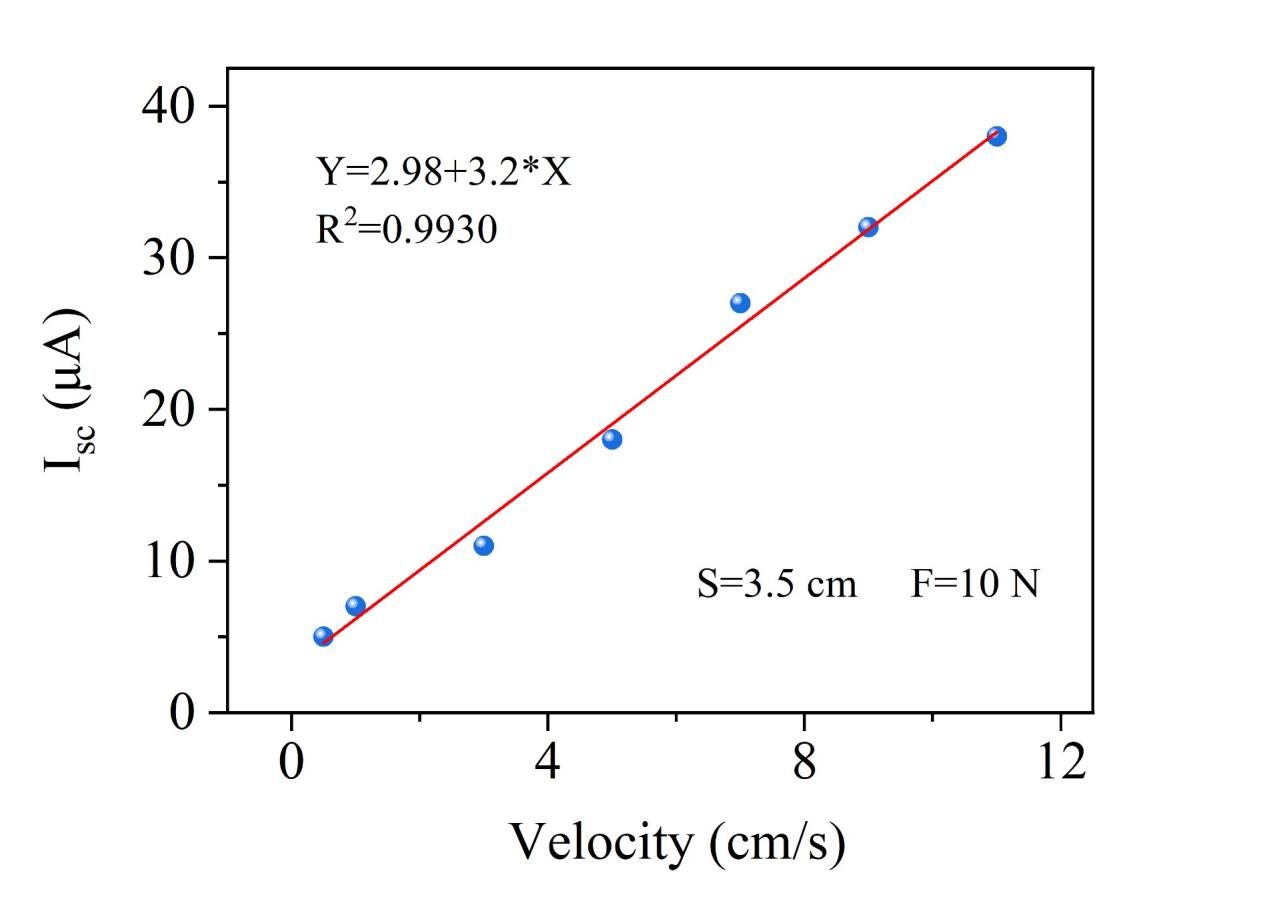
**

**Fig. S21** The fitting curve of velocity versus the short-circuit current of the WCS-TENG.

**
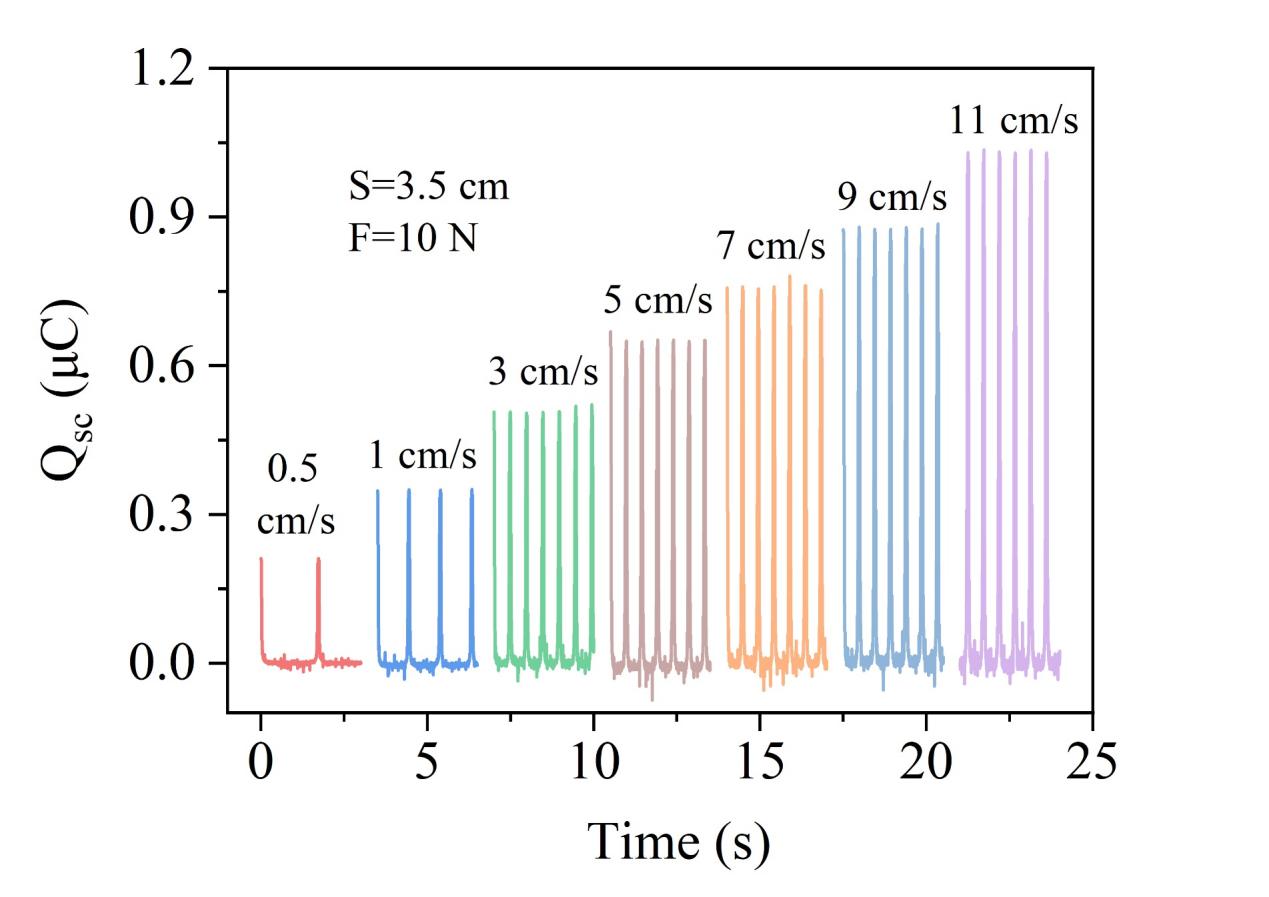
**

**Fig. S22** Effect of velocity on the transferred charge of the WCS-TENG.

**
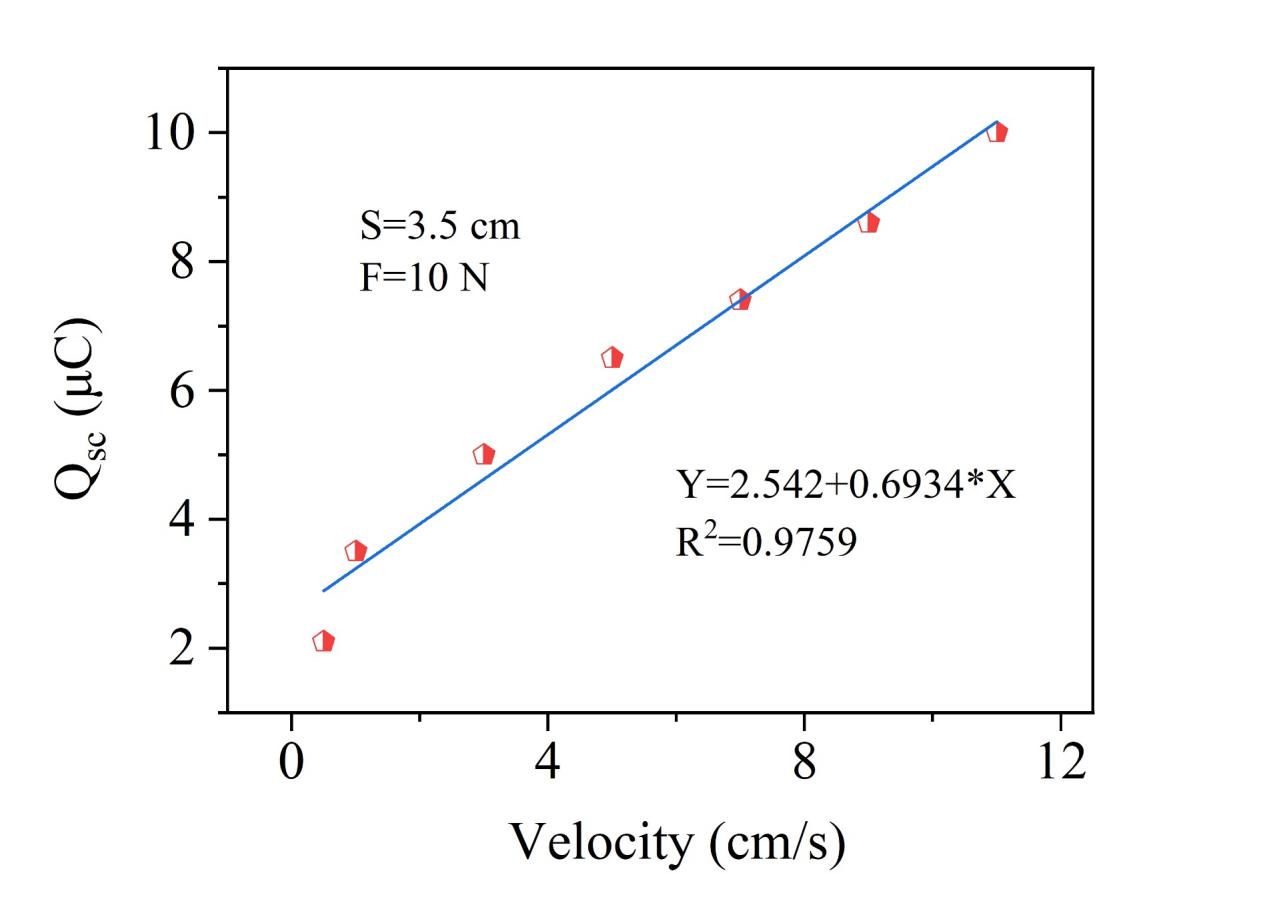
**

**Fig. S23** Fitting curve of velocity versus the transferred charge of the WCS-TENG.

**
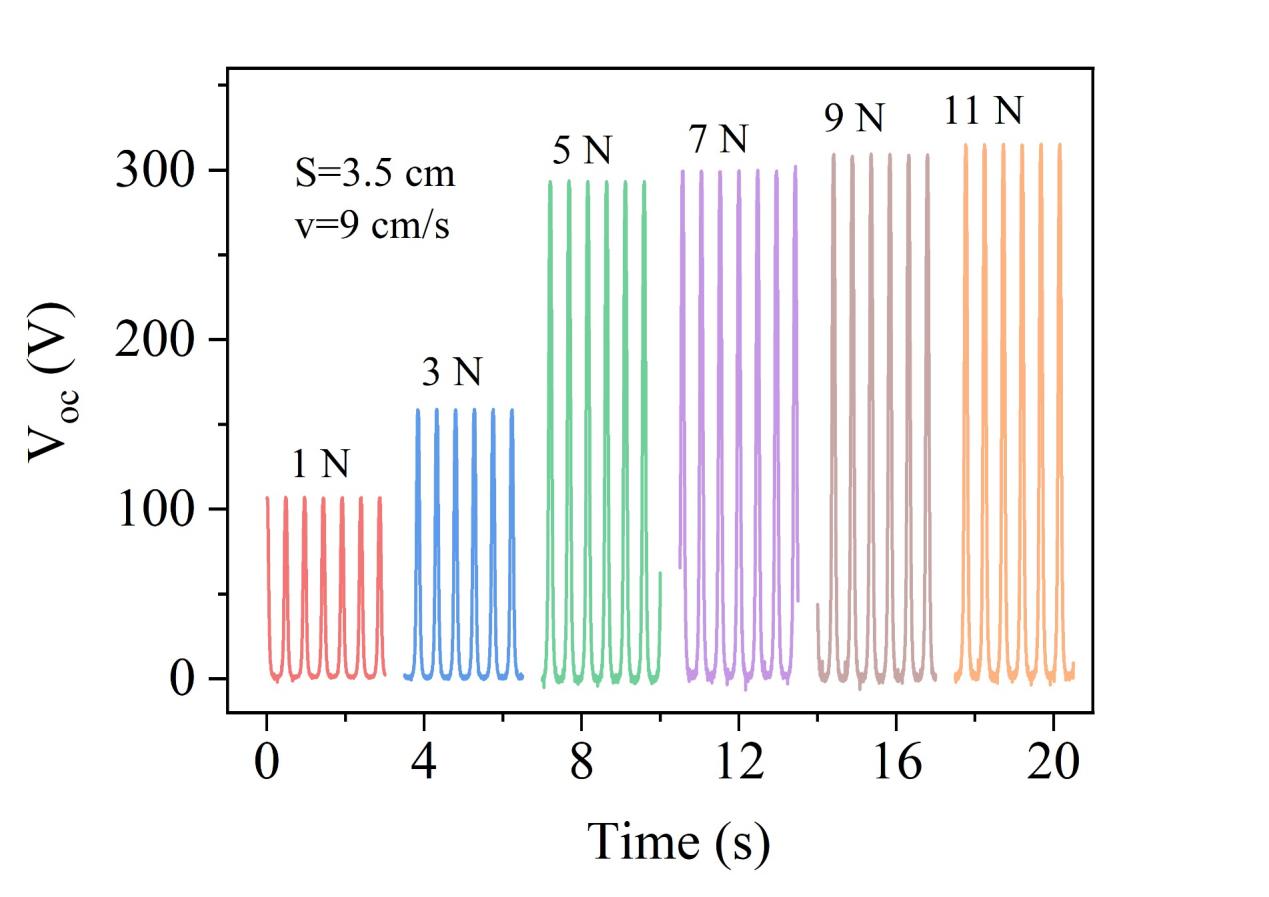
**

**Fig. S24** Effect of pressure on the open-circuit voltage of the WCS-TENG.

**
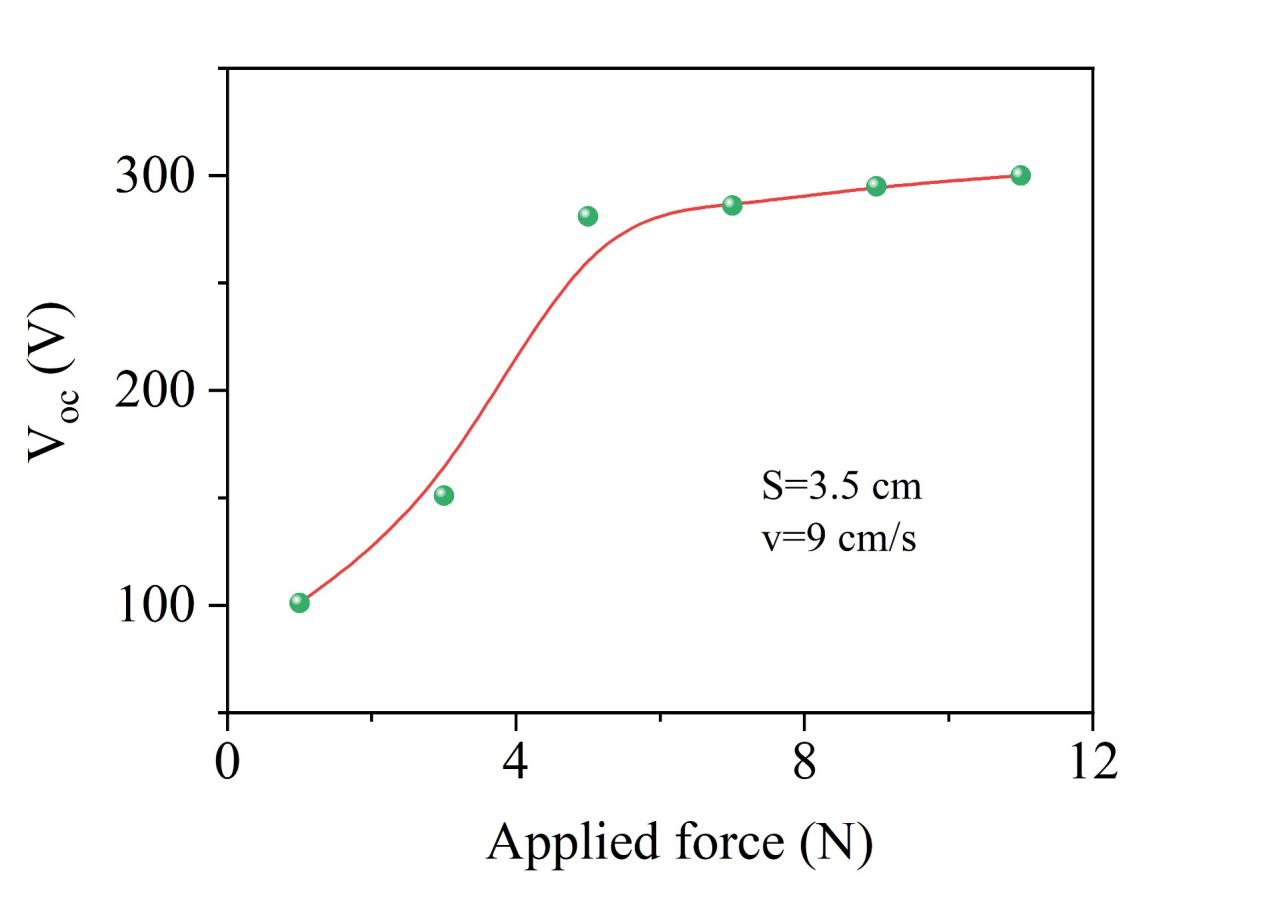
**

**Fig. S25** Fitting curve of pressure versus the open-circuit voltage of the WCS-TENG.

**
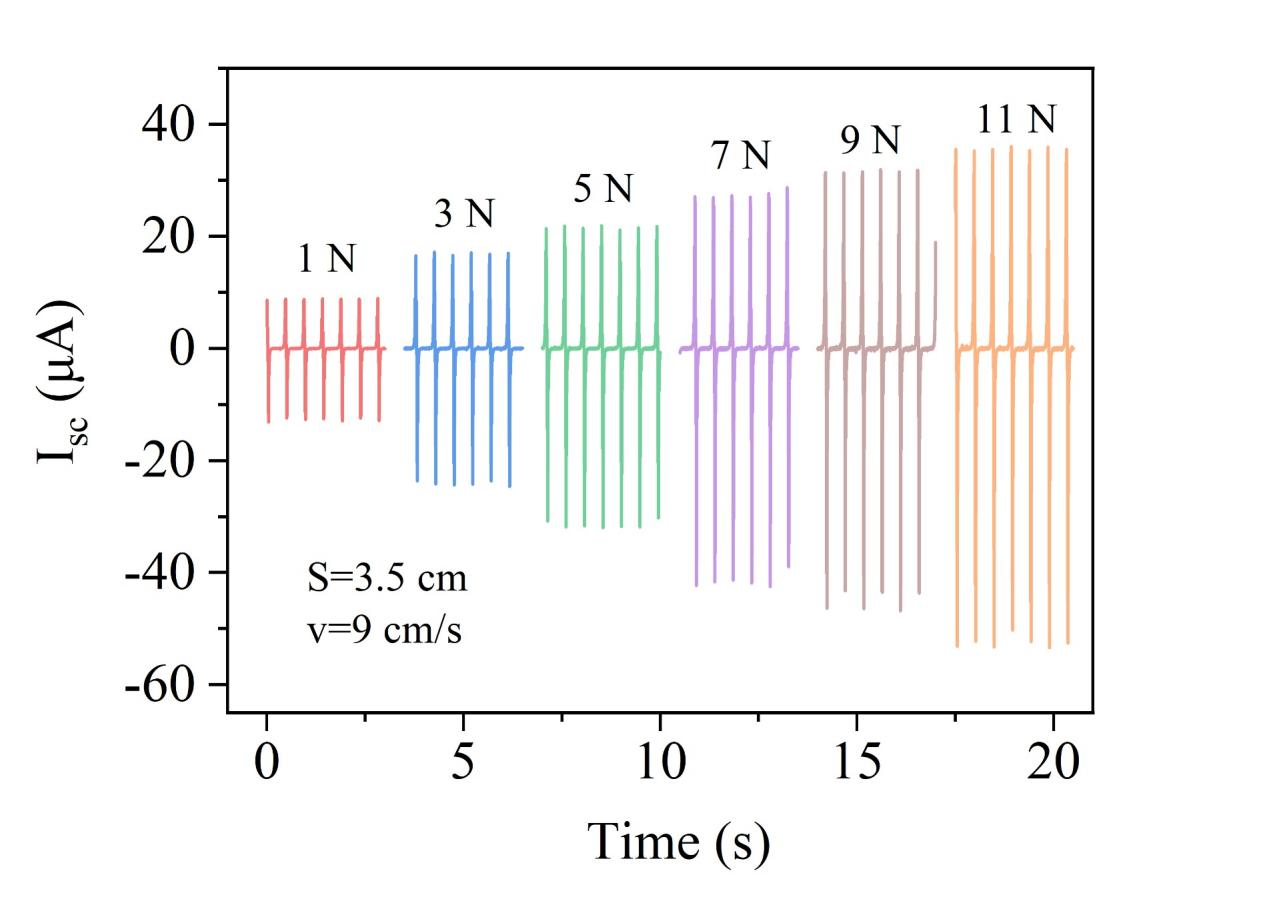
**

**Fig. S26** Effect of pressure on the short-circuit current of the WCS-TENG.

**
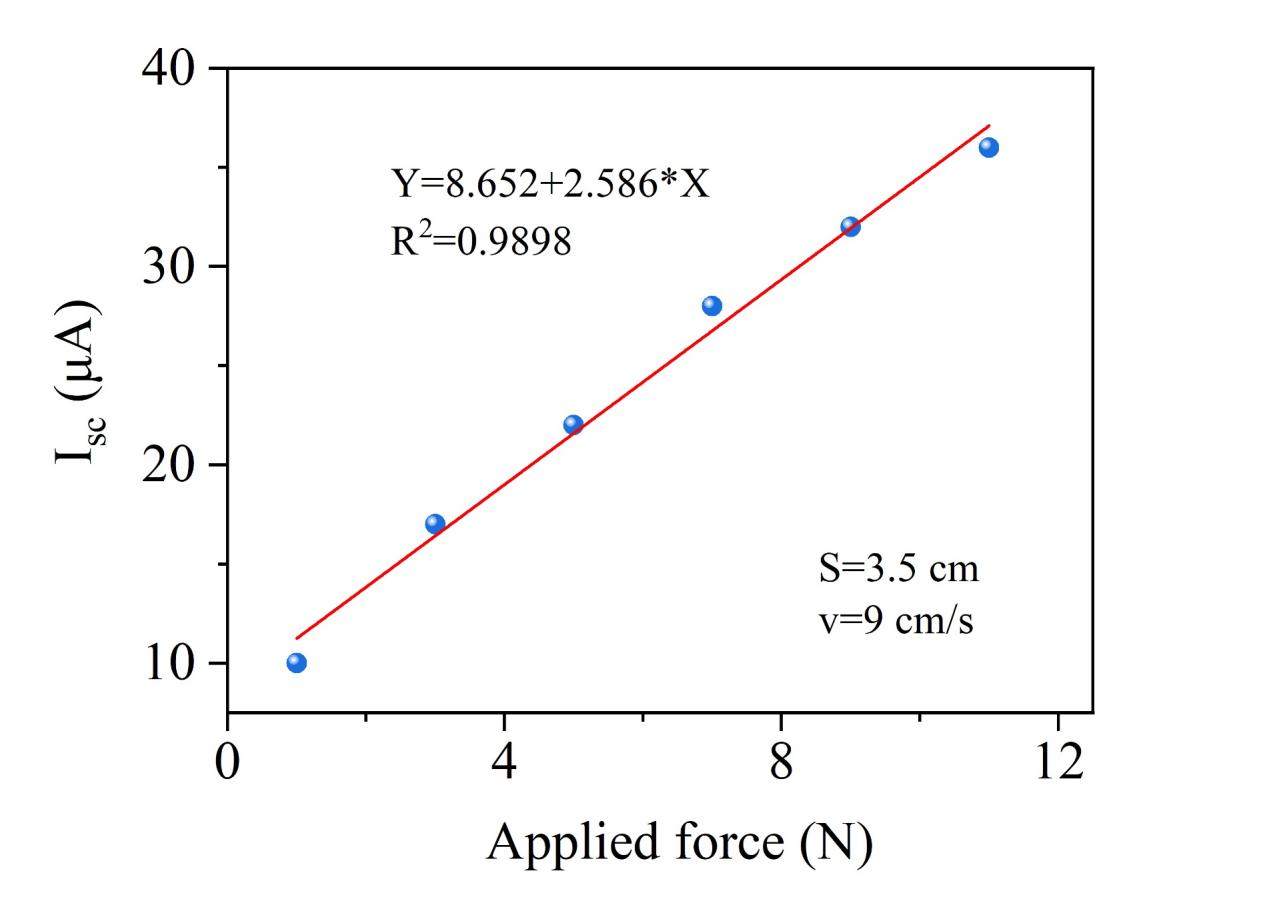
**

**Fig. S27** Fitting curve of pressure versus the short-circuit current of the WCS-TENG.

**
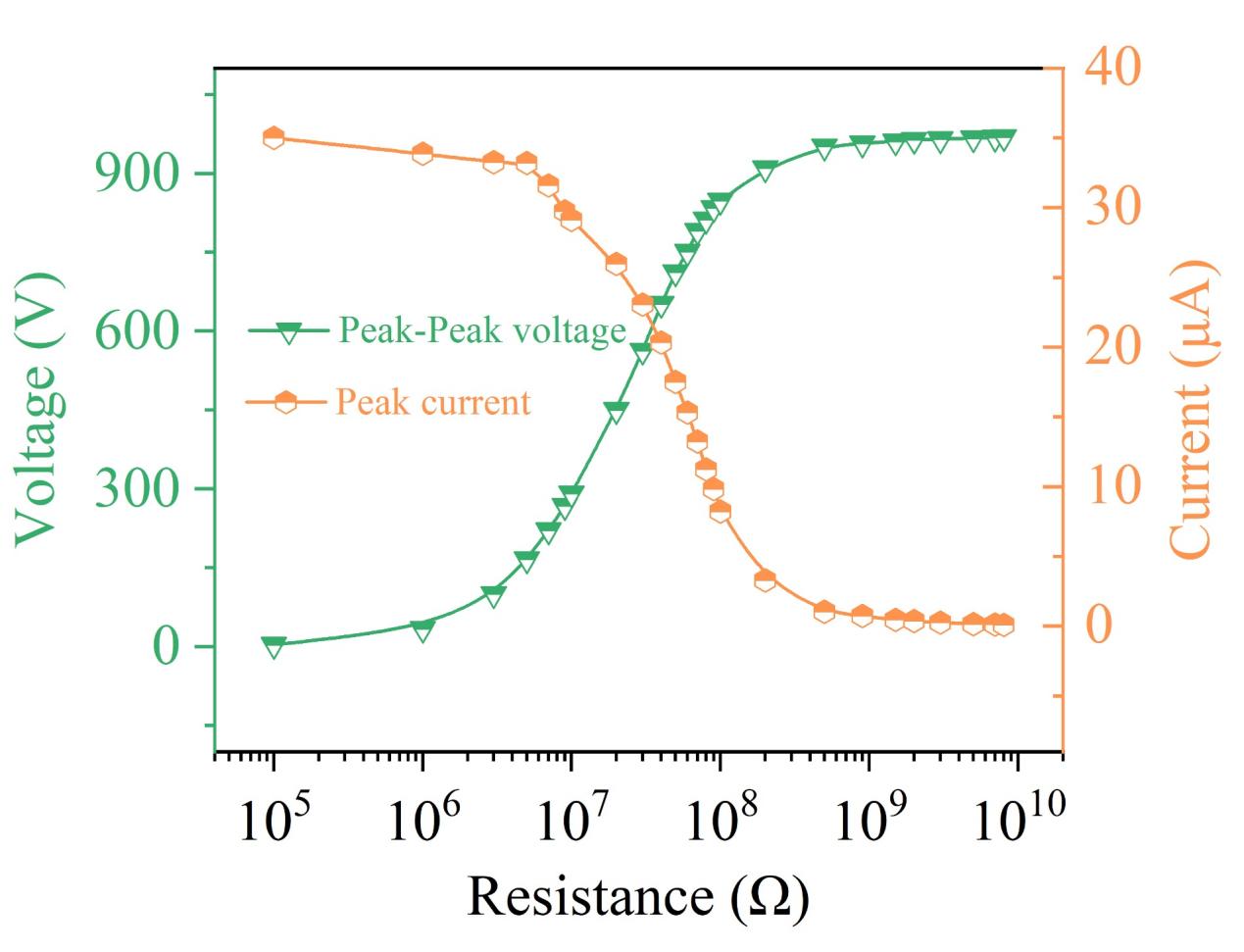
**

**Fig. S28** Variation curves of voltage and current of the WCS-TENG with external load resistance.

**
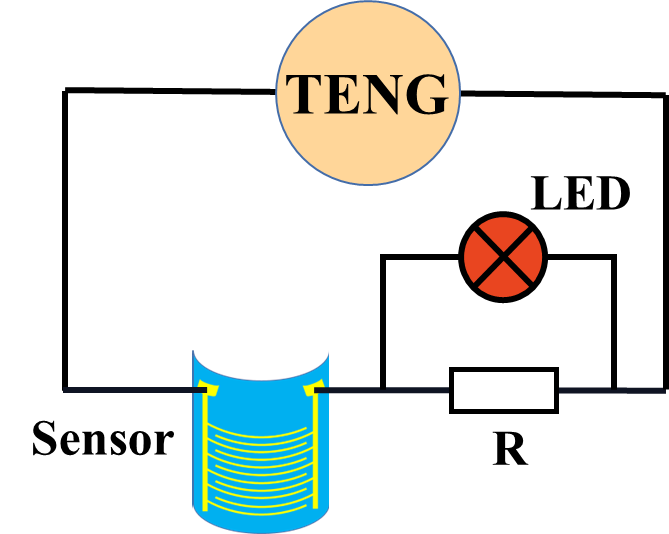
**

**Fig. S29** Schematical diagram of the alarm circuit of the self-powered NH_3_ sensor driven by the WCS-TENG.

**Table S1** EDS elemental composition analysis table of ZnO/GO nanocomposites.

| Element | Intensity (c/s) | Mass ratio (wt.%) |
| --- | --- | --- |
| C | 95.23 | 33.482 |
| N | 12.87 | 9.118 |
| O | 50.34 | 12.743 |
| Zn | 287.2 | 44.657 |

**Table S2** Comparison of repeatability of sensors fabricated from five different batches.

| Sensor Number | Concentration/  ppm | Response value/% | Response time/s | Recovery time/s |
| --- | --- | --- | --- | --- |
| 1 | 10 | 68.19 | 17 | 26 |
| 2 | 10 | 67.25 | 17 | 25 |
| 3 | 10 | 69.85 | 18 | 26 |
| 4 | 10 | 69.16 | 16 | 27 |
| 5 | 10 | 67.53 | 18 | 25 |
| Average | | 68.39 | 17.2 | 25.8 |
| Standard deviation | | 1.10 | 0.84 | 0.84 |

**Table S3** Performance comparison of our sensor with other reported ammonia gas sensors.

| Sensing material | Working temperature/℃ | Response time/s | Recovery time/s | Response /% | Detection limit/ppm | Ref. |
| --- | --- | --- | --- | --- | --- | --- |
|  |  | at 10 ppm | | |  |  |
| PANI | 25 | 193 | 449 | 200 | 5 | ^[1]^ |
| SrGe_4_O_9_/PANI | 25 | 108 | 320 | 210 | 0.2 | ^[2]^ |
| TfmpoPcCo | 25 | 23.4 | 148.5 | 0.97 | 0.05 | ^[3]^ |
| PPy NWs | 20 | 28.8 | 119.7 | 8.8 | 0.05 |  |
| MCNT | 20 | 27.0 | 57.6 | 0.38 | 0.05 |  |
| MCNT/PPy | 20 | 18.9 | 146.7 | 11 | 0.05 |  |
| TfmpoPcCo/MCNT | 20 | 219.6 | 307.8 | 0.91 | 0.05 |  |
| Bi_2_S_3_ | 20 | 25 | 70 | 450 | 1 | ^[4]^ |
| PPy-GO-WO_3_ | 20 | 50 | 120 | 58 | 5 | ^[5]^ |
| NiO/PANI | 20 | 149 | 257 | 42 | 0.5 | ^[6]^ |
| α-Fe_2_O_3_/graphene | 250 | 152 | 648 | 13 | 10 | ^[7]^ |
| In_2_O_3_/Co_3_O_4_ | 250 | 92 | 51 | 750 | 0.5 | ^[8]^ |
| Pt/ZnO/g-C_3_N_4_ | 250 | 40 | 14 | 530 | 0.5 | ^[9]^ |
| PFOTES-Ti_3_C_2_T_x_-CNF | 25 | 12 | 14 | 55 | 10 | ^[10]^ |
| BN-H/P-BNT | 25 | 65 | 25 | 250 | 1 | ^[11]^ |
| DM-PG | 25 | 110 | 650 | 42 | 0.2 | ^[12]^ |
| MXene/CuO | 25 | 43 | 26 | 120 | 1 | ^[13]^ |
| AgNW/p-SiNM | 85 | 200 | 3500 | 183 | 1 | ^[14]^ |
| ZnO-SnO_2_ | 20 | - | - | - | 50 | ^[15]^ |
| Pt/N-mWO_3_ | 260 | 18 | 40 | 300 | 5 | ^[16]^ |
| ZnO/GO | 25 | 17 | 26 | 60.76 | 0.1 | This work |

Note: “-” means not measured.

**Note S1**. Specific performance parameters of the WCS-TENG.

The experimental data demonstrated the strong linear relationship between Voc and the motion velocity, with the linear fitting equation of Y = 264.81 + 3.24*X and the goodness of fitting R^2^ = 0.9925 (Fig. S19). Simultaneously, the short-circuit current (I_sc_) peak value of the WCS-TENG increased linearly from 5 μA to 38 μA as the velocity increased (Fig. S20), with the fitting equation of Y = 2.98 + 3.2*X and R^2^ = 0.9930 (Fig. S21). The current enhancement was attributed to the higher contact frequency, which promoted the greater charge transfer rate. Furthermore, the transferred charge (Qsc) also rose with increasing velocity, with its peak value increasing from 0.2 μC to 1.03 μC. The linear fitting equation was Y = 2.542 + 0.6934*X with fitting coefficient R^2^ = 0.9759 (Fig. S22-23), indicating that the faster contact-separation process not only accelerated the charge generation but also enhanced the quantity of charge transferred per unit time. In the pressure regulation experiments (Fig. S24-25), the Voc initially increased and then saturated as the applied pressure rose from 1 N to 11 N. The behavior may result from the improved frictional contact and the increased effective contact area at low pressures, which enhanced charge generation. However, once the sufficient contact was achieved, the surface charge density reached saturation, and further pressure increments yield minimal voltage enhancement. The I_sc_ exhibited the linear increase with pressure, following the fitting equation Y = 8.652 + 2.586*X with R^2^ = 0.9898 (Fig. S26-S27), which was likely due to improved carrier transport efficiency under greater contact force, boosting instantaneous current output. To evaluate the external load response of the WCS-TENG, the voltage and current outputs under varying external resistances were measured (Fig. S28).

**Reference**

[1] L. Kumar, I. Rawal, A. Kaur, S. Annapoorni, Flexible room temperature ammonia sensor based on polyaniline, *Sensors and Actuators B: Chemical* **2017**, *240*, 408.

[2] Y. Zhang, J. Zhang, Y. Jiang, Z. Duan, B. Liu, Q. Zhao, S. Wang, Z. Yuan, H. Tai, Ultrasensitive flexible NH_3_ gas sensor based on polyaniline/SrGe_4_O_9_ nanocomposite with ppt-level detection ability at room temperature, *Sensors and Actuators B: Chemical* **2020**, *319*, 128293.

[3] S. Gai, B. Wang, X. Wang, R. Zhang, S. Miao, Y. Wu, Ultrafast NH_3_ gas sensor based on phthalocyanine-optimized non-covalent hybrid of carbon nanotubes with pyrrole, *Sensors and Actuators B: Chemical* **2022**, *357*, 131352.

[4] L. Zhou, B. Zhai, Z. Hu, M. Zhang, L. Li, X. Wang, G. Zhang, J. Luo, H. Li, B. Chen, S. Jiang, H.-Y. Li, H. Liu, Integrated sensor based on acoustics-electricity-mechanics coupling effect for wireless passive gas detection, *Nano Res.* **2023**, *16*, 3130.

[5] H. Albaris, G. Karuppasamy, Investigation of NH_3_ gas sensing behavior of intercalated PPy–GO–WO_3_ hybrid nanocomposite at room temperature, *Materials Science and Engineering: B* **2020**, *257*, 114558.

[6] Q. Hu, Z. Wang, J. Chang, P. Wan, J. Huang, L. Feng, Design and preparation of hollow NiO sphere- polyaniline composite for NH_3_ gas sensing at room temperature, *Sensors and Actuators B: Chemical* **2021**, *344*, 130179.

[7] V. Haridas, A. Sukhananazerin, J. Mary Sneha, B. Pullithadathil, B. Narayanan, α-Fe_2_O_3_ loaded less-defective graphene sheets as chemiresistive gas sensor for selective sensing of NH_3_, *Applied Surface Science* **2020**, *517*, 146158.

[8] A. N. Begi, S. Hussain, M. J. Liaqat, N. S. Alsaiari, M. Ouladsmane, G. Qiao, G. Liu, Unlocking low-concentration NH_3_ gas sensing: An innovative MOF-derived In_2_O_3_/Co_3_O_4_ nanocomposite approach, *Materials Science in Semiconductor Processing* **2024**, *181*, 108641.

[9] H. Tian, H. Fan, J. Ma, Z. Liu, L. Ma, S. Lei, J. Fang, C. Long, Pt-decorated zinc oxide nanorod arrays with graphitic carbon nitride nanosheets for highly efficient dual-functional gas sensing, *Journal of Hazardous Materials* **2018**, *341*, 102.

[10] W. Zhang, J. Zhao, C. Cai, Y. Qin, X. Meng, Y. Liu, S. Nie, Gas-sensitive cellulosic triboelectric materials for self-powered ammonia sensing, *Advanced Science* **2022**, *9*, 2203428.

[11] Q. Wang, M. Wang, K. Zheng, W. Ye, S. Zhang, B. Wang, X. Long, High-performance room temperature ammonia sensors based on pure organic molecules featuring B-N covalent bond, *Advanced Science* **2024**, *11*, 2308483.

[12] J. Qin, J. Gao, X. Shi, J. Chang, Y. Dong, S. Zheng, X. Wang, L. Feng, Z.-S. Wu, Hierarchical ordered dual-mesoporous polypyrrole/graphene nanosheets as bi-functional active materials for high-performance planar integrated system of micro-supercapacitor and gas sensor, *Adv. Funct. Mater.* **2020**, *30*, 1909756.

[13] D. Wang, D. Zhang, Y. Yang, Q. Mi, J. Zhang, L. Yu, Multifunctional latex/polytetrafluoroethylene-based triboelectric nanogenerator for self-powered organ-like MXene/metal–organic framework-derived CuO nanohybrid ammonia sensor, *ACS Nano* **2021**, *15*, 2911.

[14] J. Shin, K. Kim, I. S. Min, M. Sang, J. Y. Lee, K. Hwang, Y. Kang, J. Kim, K. J. Yu, A wireless wearable sensor system based on a silver nanowire-decorated silicon nanomembrane for precise and continuous hazardous gas monitoring, *Advanced Functional Materials* **2025**, *35*, 2419110.

[15] V. Krishnamurthi, H. Alluhaybi, P. Vashishtha, X. Guo, H. H. Nguyen, J. K. Farsani, A. Zavabeti, S. P. Giridhar, A. Jannat, S. K. Nath, A. Elbourne, S. Walia, T. Daeneke, Y. Sabri, C. K. Nguyen, N. Syed, Liquid metal printed zinc tin composite oxide nanosheets: A platform for multifunctional sensing at room temperature, *Advanced Science* *n/a*, e10017.

[16] F. Jiang, Y. Deng, K. Chen, J. Li, X.-Y. Huang, Y. Zou, L. Wu, W. Xie, Y. Deng, A straightforward solvent-pair-enabled multicomponent coassembly approach toward noble-metal-nanoparticle-decorated mesoporous tungsten oxide for trace ammonia sensing, *Advanced Materials* **2024**, *36*, 2313547.
